# Degradation Mechanisms of 4,7-Dihydroxycoumarin Derivatives in Advanced Oxidation Processes: Experimental and Kinetic DFT Study

**DOI:** 10.3390/ijerph20032046

**Published:** 2023-01-22

**Authors:** Žiko Milanović, Dušan Dimić, Erik Klein, Monika Biela, Vladimír Lukeš, Milan Žižić, Edina Avdović, Drago Bešlo, Radiša Vojinović, Jasmina Dimitrić Marković, Zoran Marković

**Affiliations:** 1 Department of Science, Institute for Information Technologies, University of Kragujevac, Jovana Cvijića bb, 34000 Kragujevac, Serbia; 2 Faculty of Physical Chemistry, University of Belgrade, 12−16 Studentski Trg, 11000 Belgrade, Serbia; 3 Institute of Physical Chemistry and Chemical Physics, Slovak University of Technology in Bratislava, Radlinského 9, SK-812 37 Bratislava, Slovakia; 4 Life Sciences Department, Institute for Multidisciplinary Research, University of Belgrade, Kneza Višeslava 1, 11030 Belgrade, Serbia; 5 Department of Agroecology and Environmental Protection, Faculty of Agrobiotechnical Sciences Osijek, University Josip Juraj Strossmayer Osijek, Vladimir Prelog 1, 31000 Osijek, Croatia; 6 Faculty of Medical Sciences, University of Kragujevac, Svetozara Markovića 69, 34000 Kragujevc, Serbia; 7 Department of Chemical-Technological Sciences, State University of Novi Pazar, Vuka Karadžića bb, 36300 Novi Pazar, Serbia

**Keywords:** 4,7-dihydroxycoumarin, DFT, EPR, AOPs, radical scavenging, hydroxyl radical, QM−ORSA

## Abstract

Coumarins represent a broad class of compounds with pronounced pharmacological properties and therapeutic potential. The pursuit of the commercialization of these compounds requires the establishment of controlled and highly efficient degradation processes, such as advanced oxidation processes (AOPs). Application of this methodology necessitates a comprehensive understanding of the degradation mechanisms of these compounds. For this reason, possible reaction routes between HO^•^ and recently synthesized aminophenol 4,7-dihydroxycoumarin derivatives, as model systems, were examined using electron paramagnetic resonance (EPR) spectroscopy and a quantum mechanical approach (a QM-ORSA methodology) based on density functional theory (DFT). The EPR results indicated that all compounds had significantly reduced amounts of HO^•^ radicals present in the reaction system under physiological conditions. The kinetic DFT study showed that all investigated compounds reacted with HO^•^ via HAT/PCET and SPLET mechanisms. The estimated overall rate constants (*k*_overall_) correlated with the EPR results satisfactorily. Unlike HO^•^ radicals, the newly formed radicals did not show (or showed negligible) activity towards biomolecule models representing biological targets. Inactivation of the formed radical species through the synergistic action of O_2_/NO*_x_* or the subsequent reaction with HO^•^ was thermodynamically favored. The ecotoxicity assessment of the starting compounds and oxidation products, formed in multistage reactions with O_2_/NO*_x_* and HO^•^, indicated that the formed products showed lower acute and chronic toxicity effects on aquatic organisms than the starting compounds, which is a prerequisite for the application of AOPs procedures in the degradation of compounds.

## 1. Introduction

The rapid advancement of synthetic organic chemistry has led to the appearance of numerous compounds with remarkable biological properties. Available literature data indicate that various newly synthesized coumarin derivatives have pronounced pharmacological and biological properties, such as anticarcinogenic [1,2], antimicrobial [3,4], antioxidant [5,6], antiviral [7,8], and, especially, anticoagulant activities [9,10]. Coumarin derivatives, such as warfarin and coumatetralyl, are some of the most frequently used anticoagulant agents [11]. On the other hand, the mentioned compounds are also potent rodenticides that act through the same anticoagulant mechanism [12]. Due to their pharmaceutical and industrial applications, significant concentrations of these compounds and their hydroxylated derivatives are present in wastewater treatment plants, from where they often end up in natural receiving waters as part of effluents [13,14]. Due to their stability and persistence, coumarin derivatives reabsorbed in the diets of aquatic organisms can be especially dangerous. Their pronounced biological activity and the potential application of newly synthesized coumarin derivatives in industry have resulted in the need for a specific, highly efficient methodology for the study of their degradation. In recent decades, particular emphasis has been placed on the application of techniques based on advanced oxidation processes (AOPs) [15,16]. The first step in AOPs is the in situ generation of strong oxidants—e.g., through the Fenton reaction or photocatalytic oxidation—capable of oxidizing various compounds [17,18]. However, the application of this methodology requires the development of a strategy and modeling of the reaction process. This necessitates a comprehensive investigation of the mechanisms of the reaction between the highly reactive radical species and the corresponding compound, as well as an assessment of the toxicity of the formed intermediates [19]. Reliable knowledge about the possible mechanisms, intermediates, and final products is crucial for successful modeling of AOPs. Standard experimental techniques are often limited in providing unambiguous identification of mechanisms due to the formation of unstable intermediates that are difficult to detect [20].

**Figure 1:**
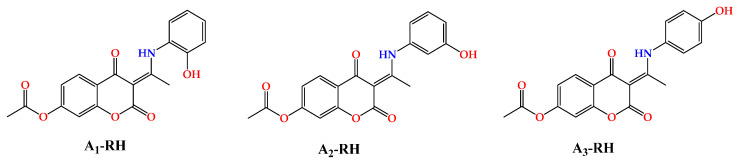
Structures of previously synthesized [21] aminophenol derivatives of 4,7−dihydroxycoumarin.

For this reason, the degradation mechanisms of previously synthesized aminophenol 4,7-dihydroxycoumarin derivatives (Figure 1) [21] were examined in this study under AOP conditions (HO^•^) as examples of stable aromatic compounds utilizing the sophisticated Electron paramagnetic resonance (EPR) spectroscopy experimental technique and a theoretical quantum mechanics-based test for overall radical scavenging activity (the QM-ORSA methodology) [22] based on density functional theory (DFT). The structure of these compounds offers the possibility of analyzing the effect of the substituent (–OH) position on the reaction parameters. A similar methodology has been successfully applied to other coumarin derivatives [23,24].

The applied methodology was based on the calculation of the thermodynamic and kinetic parameters of generally accepted radical scavenging mechanisms, such as hydrogen atom transfer (HAT), single-electron transfer followed by proton transfer (SE−TPT), sequential proton loss followed by electron transfer (SPLET), and radical adduct formation (RAF) [25,26]. In addition, assessment of the reactivity of the newly formed radical species utilizing appropriate models for biomolecular targets was one of the main goals of this work, since one of the prerequisites for AOPs is the lower reactivity of final products. A theoretical prediction of the ecotoxicity of the formed products toward aquatic organisms was also calculated using available software resources. The obtained results for the title compounds, as model systems, represent a basis for future investigations into the degradation processes of different coumarin derivatives.

## 2. Materials and Methods

### 2.1. Chemicals and Instrumentations

Chemicals used in the synthesis and spectroscopic EPR measurements were obtained from Merck (Darmstadt, Germany), except for spin-trap DEPMPO, which was purchased from Enzo Life Sciences (Farmingdale, NY, USA). 

### 2.2. EPR Measurement with HO^•^ Radical

The Bruker EMX Nano X-band (9.65 GHz) spectrometer was used for the EPR measurements, which were conducted at room temperature (293 K) using the following experimental parameters: 10 dB power attenuation; 2 mT modulation amplitude; 100 kHz modulation frequency; and 120 s sweep time. The hydroxyl radical (HO^•^) was generated in 100 mM phosphate buffer, pH = 7.4, using the standard Fenton reaction (1 mM H_2_O_2_ and 0.33 mM FeSO_4_) with the addition of 0.1 M 2-diethoxyphosphoryl-2-methyl-1-oxido-3,4-dihydropyrrol-1-ium (DEPMPO) as a spin-trapping agent. Spectra collection started 180 s after the addition of the iron catalyst. Jackson’s procedure for purifying the spin trap was followed [27]. Stock solutions of compounds (15 mM) were prepared in DMSO and diluted to 10 μM with water. The amount of DMSO in the blank sample was the same as in the samples containing the investigated compounds. The final concentration of the examined compounds was 0.75 μM. The average intensity of the two most intense peaks of the DEPMPO–HO^•^ adduct at the low-field region of the spectrum was used to calculate reactivities to HO^•^. Measurement results are expressed as the % of radical reduction = 100 × (*I*_0_ − *I*_a_)/*I*_0_. In the previous equation, *I*_a_ and *I*_0_ are the intensities of the peaks of the DEPMPO–HO^•^ adduct with and without the investigated compounds (A_1_–RH–A_3_–RH), respectively. 

### 2.3. Computational Methodology

The Gaussian09 program package [28] was used for all the calculations based on density functional theory (DFT). The M06-2X/6-311++G(d,p) theoretical model (with polarization and diffuse functions included) was employed for the optimization of the structures of the coumarin derivatives, as suggested in [29]. The applied theoretical model is suitable for thermodynamic and kinetic analyses of various reactions [23,24,25,30,31]. The conductor-like polarizable continuum model (CPCM, water (*ε* = 78.36)) was applied to approximate the solvent effect in the experimental environment [32].

The radical mechanisms presented in this study were evaluated based on thermodynamic and kinetic considerations. This was consistent with the quantum mechanics-based test for overall free radical scavenging activity (QM-ORSA) methodology [22], commonly used to determine antiradical activity. After the calculation of the corresponding reaction Gibbs free energies (Δ_r_*G*), kinetic calculations were performed for all exergonic (Δ_r_*G* < 0) and isoergonic (Δ_r_*G* = 0) reaction pathways. The rate constants (*k*) were calculated using transition state theory (TST) [33] or the Eyring equation, as well as Eckart’s method, which represents the special case of the zero-curvature tunneling approach (ZCT_0) [34]. The first theory is based on the laws of classical kinetics, whereas the second includes quantum effects, such as tunneling (Equation (1)):

(1) $$k_{ZCT\_0}=\sigma \gamma (T)\frac{k_{B}T}{h}\exp\left(\frac{-\Delta G_{a}^{\neq }}{RT}\right)$$

where *k*_B_ and *h* are the Boltzmann and Planck constants; *T* is the temperature in K (298.15 K); Δ*G*_а_^≠^ is the activation Gibbs free energy; *σ* represents the reaction path degeneracy accounting for the number of equivalent reaction paths; and *γ*(*T*) is the tunneling correction [35]. For these calculations, *TheRate* program was used [36].

Evaluation of the overall rate constant (*k*_overall_) in a polar medium offers a comprehensive picture of the reactivity of the investigated compounds. The *k*_overall_ is the sum of the products of the molar fractions of acid–base species included in specific reactions and the total rate constant (*k*_tot_). The *k*_tot_ comprises the sum of all kinetically favored reaction pathways for a particular species. A detailed explanation of the *k*_overall_ estimation, the process of quantifying molar fractions of acid–base species at physiological pH, is given in previous research [23]. Additionally, the equations for the estimation of reactivity towards a specific radical (*r^T^*) relative to the reference standard antioxidant (Trolox), as well as relative amounts of products (%)—i.e., the branching ratios (*Г_i_*)—are integral parts of a previous report [23]. 

The Ecological Structure–Activity Relationships program (ECOSAR V2.0) [37] was used to evaluate the acute and chronic toxicities (ChV, mg·L^−1^) of the investigated compounds and their oxidation products towards aquatic organisms: green algae, fish, and daphnia. Acute toxicity was defined using EC_50_ values (the concentration of the examined compound that affected the growth of 50% of green algae after 96 h of exposure) and LC_50_ values (the concentration of the investigated compound that caused 50% mortality in fish and daphnia after 96 h) [38,39].

The estimated *k*_overall_ values made it possible to determine the stability of the investigated compounds during their degradation initiated by HO^•^ radicals through the half-life (*τ*_1/2_) using the following equation:

(2) $$\tau_{1/2}=\ln2/k_{\mathrm{overall}}\times \left[HO^{•}\right]$$

where [HO^•^]_aq_ is the concentration of HO^•^ in an aqueous solution [40].

**Figure 2:**
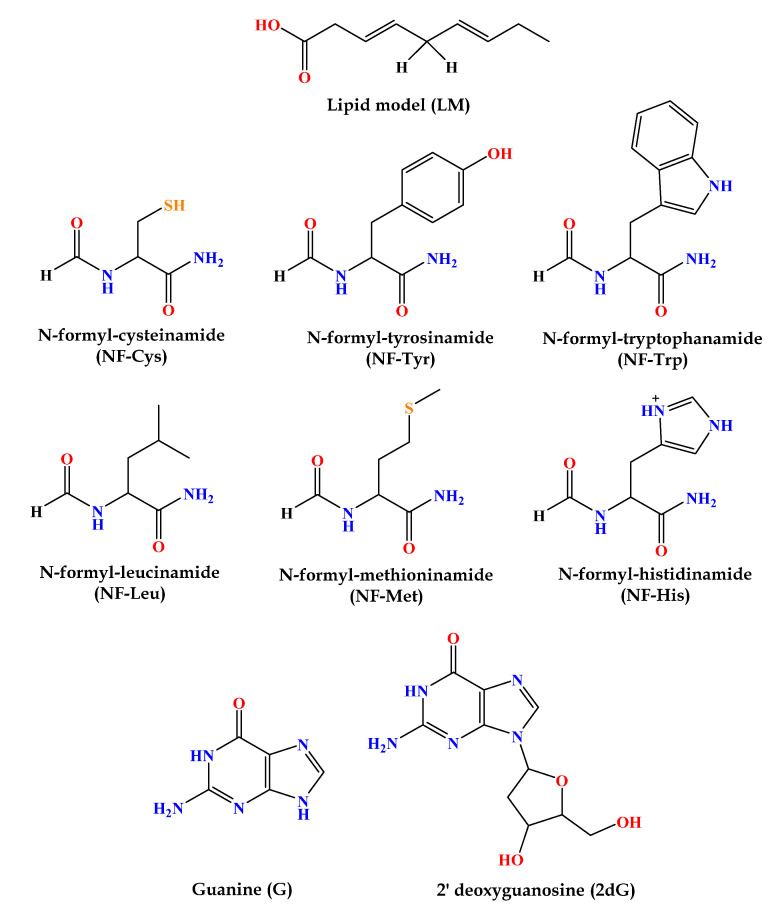
Structures of investigated molecular targets—constituents of important molecules: phospholipid bilayer, proteins, and nucleic acids.

To examine the activity of the newly formed radical products (**A_1_–R^•^**, **A_2_–R^•^**, **A_3_–R^•^**) towards biologically essential macromolecules, interactions with three groups of building blocks were considered: model lipids, amino acid residues, and nucleobases, as depicted in Figure 2 [41]. The lipid model (LM) mimics unsaturated fatty acids as essential biomolecules. It is represented as a reduced linoleic acid (LA) model that retains its primary chemical reactivity characteristic: two allylic H atoms. Amino acids, as constituents of proteins, are modeled realistically. This model has been successfully used and is widely accepted as appropriate for investigating protein site reactions. The following residues, being the most susceptible to oxidative damage in proteins, were used in this study: cysteine (Cys), leucine (Leu), tyrosine (Tyr), tryptophan (Trp), methionine (Met), and histidine (His). 2′-Deoxyguanosine (2dG) was selected as a model for oxidative DNA damage because guanine (G) is the most easily oxidized nucleobase. Therefore, when one-electron oxidation of DNA occurs, it is primarily located at G sites. Consequently, if a chemical oxidant (radical species) can oxidize 2dG, it can cause oxidative damage to DNA. In contrast, if there is no potential to oxidize 2dG, the oxidant is considered harmless to DNA.

## 3. Results and Discussion

### 3.1. Experimental HO^•^ Scavenging Activity

**Figure 3:**
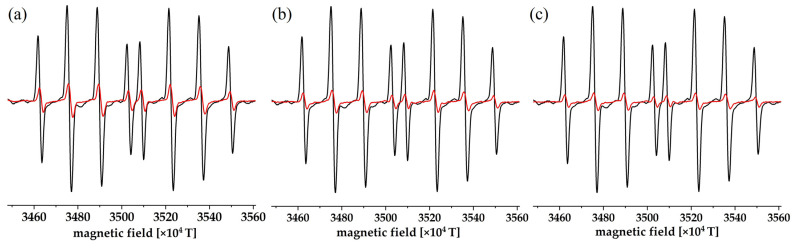
The experimental EPR spectra of DEPMPO−HO^•^ in the presence (red line) and absence (black line) of the compounds (**a**) A_1_–RH, (**b**) A_2_–RH, and (**c**) A_3_–RH.

EPR spectroscopy was used to trace the reactivity of the obtained compounds toward HO^•^. All spectra were collected starting from the same time point—180 s after the reaction beginning. Scavenged HO^•^ radicals were formed in the Fenton system, and DEPMPO was used as a spin trap to enable the monitoring of the decrease in the HO^•^ concentration. Figure 3 shows the EPR spectra of DEPMPO–HO^•^ adducts before and after the addition of A_1_–RH to A_3_–RH compounds. Signal intensity, proportional to the number of scavenged radical species, was reduced after addition of coumarin derivatives, indicating the reaction between the investigated compounds and HO^•^. The reactivity of the investigated compounds towards HO^•^ was calculated as explained in the Materials and Methods section. The scavenging activities decreased in the following order: A_1_–RH (91%) > A_2_–RH (88%) > A_3_–RH (81%). Differences in these values indicate the variation in the reactivity of the investigated compounds. The studied coumarin derivatives contained the –OH group in various positions relative to the –NH– group and the rest of the molecule, leading to different reactivities. This group made hydrogen atom/proton abstraction possible in the standard examination of the activity towards radicals. The amino group was not considered a potential hydrogen atom/proton donor, as this hydrogen atom encloses a quasi-six-membered ring through a hydrogen bond with the carbonyl group, as previously observed in the crystal structure of similar compounds [42,43,44]. The most reactive compound was A_1_–RH, which can be explained by the possible formation of hydrogen bonds between oxygen and the NH group upon the reaction with HO^•^. Due to the existence of a negative charge in the aromatic ring, higher reactivity for A_3_–RH was expected in comparison to A_2_–RH. Radical adduct formation (RAF) is another plausible mechanism, as these compounds contain many unsaturated bonds [45,46]. The following sections include a detailed quantum chemical analysis of the oxidation process, emphasizing the role of acid-base equilibria. Hydrogen atom transfer (HAT), the direct exchange of protons followed by the transfer of electrons from formed anions, and the formation of radical adducts are the most probable reaction pathways for coumarin derivatives [23,24]. These mechanisms play crucial roles in the synergy between the elimination of radical species from wastewater and the production of less harmful oxidation products [47,48,49,50].

### 3.2. Acid-Base Equilibria

**Figure 4:**
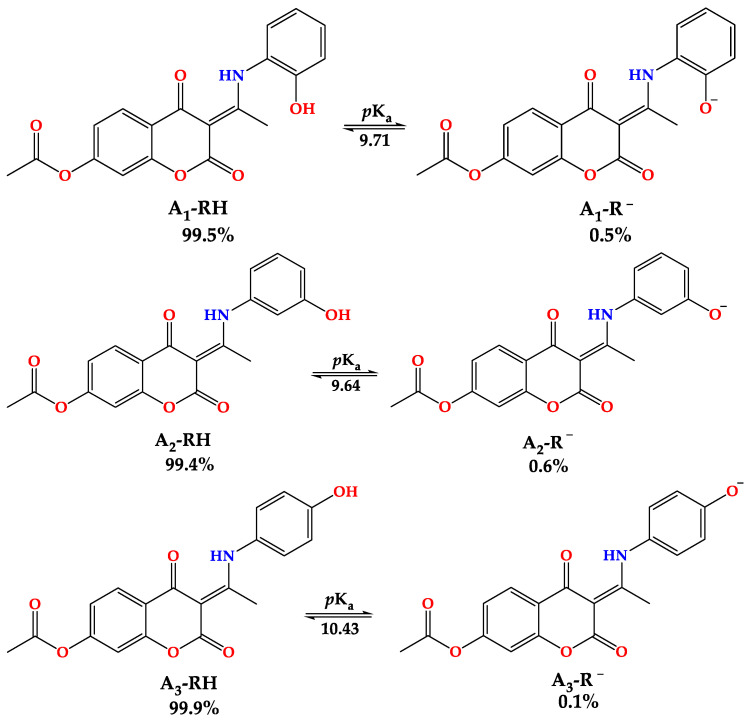
Deprotonation process, estimated *p*K_a_ values, and molar fractions (*f*) of acid–base species of newly synthesized aminophenol derivatives of 4,7−dihydroxycoumarin (A_1_RH to A_3_RH) at physiological pH = 7.4.

As the acid–base equilibrium determines the relative abundance of protonated/deprotonated forms present in an aqueous solution, it is evident that the pH value of the solution determines the dominant mechanism of free radical scavenging. The degree of deprotonation of a compound, expressed through the corresponding *p*Ka value, determines various physicochemical properties, such as hydrophobicity, lipophilicity, polarizability, etc. Quantifying the molar fractions of acid–base species provides a comprehensive way of examining the mechanisms of radical scavenging action. Therefore, it was necessary to determine the *p*Ka values to obtain the deprotonation route and quantify the molar fractions (*f*). The ACD/*p*K_a_ software package was employed to calculate the *p*K_a_ values of studied derivatives [51]. Figure 4 shows their deprotonation routes, as well as the estimated *p*Ka values and molar fraction (*f*) values under physiological conditions. The *p*K_a_ values depend on the position of the –OH group. As there were no additional stabilization effects in the formed anion, the *meta-*substituted derivative (**A_2_–R^−^**) showed the lowest value (*p*K_a_ = 9.64). The **A_1_–R^−^** anion (*p*K_a_ = 9.71) was stabilized by the intramolecular hydrogen bond between the amino group and the oxygen atom. The extended delocalization in the aromatic ring of the formed anion **A_3_–R^−^** was responsible for the highest *p*K_a_ value (*p*K_a_ = 10.43). This analysis proves that hydrogen atom/proton donation is only possible from the –OH group.

**Figure 5:**
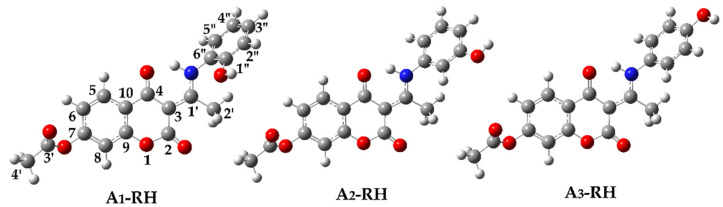
Optimized geometries of investigated compounds in water at M06-2X/6-311++G(d,p) level of theory with carbon atom numbering scheme.

Based on the obtained p*K*_a_ values, neutral species dominate (>99%) at physiological pH. Due to this fact, theoretical investigations were performed on neutral forms: A_1_–RH (99.5%), A_2_–RH (99.4%), and A_3_–RH (99.9%). Their optimized structures, with carbon atom numbering schemes, are shown in Figure 5.

### 3.3. Reactions of A_n_–RH with HO^•^ Radical—Thermodynamic Approach

The possible reaction centers for the standard mechanisms (HAT/PCET (Equation (3)), SPLET (Equations (4) and (5)), and SETPT (Equations (6) and (7))) of radical action between A_1_–RH, A_2_–RH, A_3_–RH, and HO^•^ were aromatic –OH groups. For the RAF mechanism, these centers included aromatic carbon atoms (Equation (8)). The calculated values of the reaction Gibbs energies (Δ_r_*G*) for the mentioned mechanisms are listed in Table 1.

HAT: A*_n_*–RH + HO^•^ → A*_n_*–R^•^ + H_2_O (3)

SPL: A*_n_*–RH + HO^−^ → A*_n_*–R^−^ + H_2_O (4)

ET: A*_n_*–R^−^ + HO^•^ → A*_n_*–R^•^ + HO^−^ (5)

SET: A*_n_*–RH + HO^•^ → A*_n_*–RH^•+^ + HO^−^ (6)

PT: A*_n_*–RH^•+^ + HO^−^ → A*_n_*–R^•^ + H_2_O (7)

RAF: A*_n_*–RH + HO^•^ → [HO–A*_n_*–RH]^•^ (8)

According to the Δ_r_*G* values shown in Table 1 for the first step of each mechanism, HAT/PCET was thermodynamically favored for all derivatives. The reactivity of the compounds and the stability of the formed radical products increased in the following order: A_2_–RH (−124 kJ·mol^−1^) > A_3_–RH (−126 kJ·mol^−1^) > A_1_–RH (−127 kJ·mol^−1^). This order nicely follows the discussion on the possible stabilization effects of proton removal in acid-base equilibrium processes. 

Negative Δ_r_*G*_RAF_ values made the RAF mechanism thermodynamically spontaneous in almost all the positions of the investigated compounds. The most favored positions for attack by electrophilic HO^•^ were the C5 (from −43 to −37 kJ·mol^−1^) and C7 (from −44 to −40 kJ·mol^−1^) atoms of the aromatic part of the chroman ring, as well as the C1″ to C6″ positions of the aromatic aminophenol rings. As mentioned, the aromatic carbon atoms of both rings were possible reaction sites. It is essential to notice that the values for the chroman part of the molecule did not significantly depend on the position of the substituent (around 5 kJ·mol^−1^ difference), with the exception of position C3. When aminophenol carbon atoms were involved, noticeable differences were only obtained for the carbon atoms adjacent to the position of the –OH group. However, slightly endergonic values for the C10 position of the A_1_–RH (2 kJ·mol^−1^) and A_3_–RH (4 kJ·mol^−1^) compounds were obtained. In the optimized geometries of radical adducts (Figures S1–S3), the rehybridization of the carbon atom (*sp^2^* to *sp^3^*) where the HO^•^ radical was attached occurred, leading to broken aromaticity and planarity in the system. The most thermodynamically favored products were characterized by short interatomic distances in adducts C−2″ (A_1_–RH, 1.408 Å), C−3″ (A_2_–RH, 1.405 Å), and C−5″ (A_3_–RH, 1.405 Å) due to stabilization by intramolecular contacts (Figures S1–S3).

For all the examined compounds, significantly negative Δ_r_G_SPL_ values indicated that the first step of the SPLET mechanism was thermodynamically spontaneous (Table 1). The reactivity of the compounds and the stability of the formed anionic species increased in the following sequence: A_2_–RH (−103 kJ·mol^−1^) > A_1_–RH (−115 kJ·mol^−1^) > A_3_–RH (−115 kJ·mol^−1^), with the same plausible explanation as for the first mechanism. Comparison of Δ_r_*G*_HAT/PCET_ and Δ_r_*G*_SPL_ indicated that the hydrogen atom transfer from the –OH group was slightly more favored than the proton transfer. In the second step of the SPLET mechanism—i.e., electron transfer—Δ_r_*G*_ET_ values decreased in the following sequence: A_1_–RH (−13 kJ·mol^−1^) > A_2_–RH (−21 kJ·mol^−1^) > A_3_–RH (−28 kJ·mol^−1^). These values depended on the spin delocalizations in the formed radicals.

Finally, highly endergonic values (122–144 kJ·mol^−1^) for the first step of the SET−PT mechanism (Δ_r_*G*_SET_) suggested that this mechanism was not thermodynamically probable. Thus, it was not considered in further kinetic studies (Table 1).

### 3.4. Reactions of A_n_–RH with HO^•^ Radical—Kinetic Approach

Thermodynamically favored reaction pathways (Δ_r_*G* ≤ 0) were subjected to kinetic investigation. After locating the transition state geometries (where possible), the activation Gibbs energies (Δ*G*_a_) were evaluated. Rate constants for reactions involving electron transfer were calculated using Marcus theory (Table 2). The rate constants were estimated using the TST (Table S1) and ZCT_0 (Table 2) methods. 

The pronounced exergonic values for hydrogen atom transfer (HAT/PCET) between A*_n_*–RH and HO^•^ indicate the thermodynamic favorability of this mechanism. However, attempts to find transition state geometries describing these reactions have been unsuccessful. Thus, it is reasonable to assume that these reactions occur in a practically barrier-less manner [25]. To confirm the above assumption, the energy change as a function of the corresponding distance—i.e., HO–H2″ (−OH, Å) (A_1_–RH), HO–H3″ (−OH, Å) (A_2_–RH), HO–H4″ (−OH, Å) (A_3_–RH)—was monitored (Figure S4). Analyzing Figure S4, it was discovered that there was a constant decrease in total energy as a function of distance from −1314.79 to −1314.87 a.u. This means that the reaction takes place without an activation barrier as a diffusion-controlled process with the rate constant, based on available literature data, of 1.91 × 10^9^ M^−1^·s^−1^ [52,53,54].

**Figure 6:**
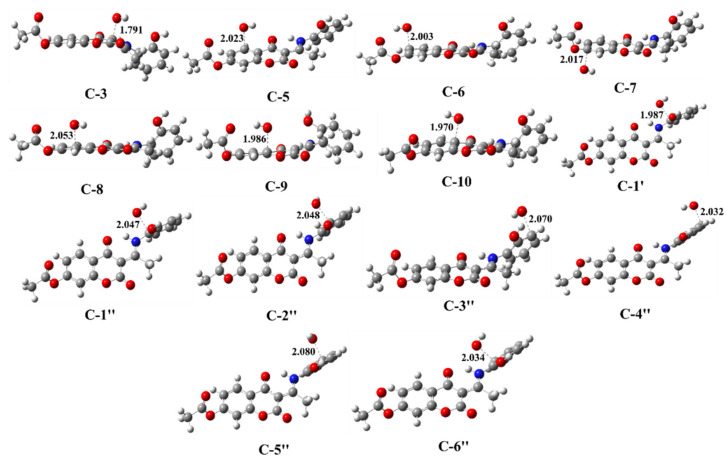
Optimized transition state geometries for radical adduct formation for A_2_–RH and HO^•^ in water at M06-2X/6−311++G(d,p) level of theory.

Another plausible mechanistic pathway for the reaction of HO^•^ with the investigated compounds is the RAF mechanism. The values of the rate constants estimated with the ZCT_0 method (Table 2) correlate with the values estimated with the TST method (Table S1). The rate constants obtained were in the range of 10^3^ to 10^7^ M^−1^·s^−1^. Comparison of thermodynamic and kinetic parameters provided evidence that thermodynamically favored products are not necessarily kinetically preferred. The kinetically most preferred positions for HO^•^ attack were C1″ (1.29 × 10^7^ M^−1^·s^−1^) and C3″ (1.20 × 10^7^ M^−1^·s^−1^) for A_1_–RH, C2″ (4.46 × 10^7^ M^−1^·s^−1^) and C4″ (1.39 × 10^8^ M^−1^·s^−1^) for A_2_–RH, and C3″ (2.44 × 10^7^ M^−1^·s^−1^) and C5″ (2.55 × 10^7^ M^−1^·s^−1^) for A_3_–RH, with Δ*G*_a_ values in the interval from 36 to 41 kJ·mol^−1^. The optimized geometries of the corresponding transition states are shown in Figure 6, Figures S5 and S6. The transition states of the kinetically most favored products were characterized by larger interatomic distances: C1″ (2.047 Å) and C3″ (2.070 Å) for A_1_–RH, C2″ (2.093 Å) and C4″ (2.115 Å) for A_2_–RH, and C3″ (2.077 Å) and C5″ (2.088 Å) for A_3_–RH. Moreover, the geometries of the mentioned transition states were stabilized by hydrogen bonds between the reactive HO^•^ particle and the polar functional group.

Analogously to the HAT/PCET mechanism, the geometries of the transition states for the proton transfer reactions (SPL mechanism) have not been found, despite numerous attempts. In this case, the dependence of the total energy (a.u.) on the distance was monitored. The constant decrease in energy indicated that these reactions occurred without an activation barrier as diffusion-controlled processes (Figure S7, 1.91 × 10^9^ M^−1^·s^−1^) [52,53,54]. The electron transfer rate constants estimated using Marcus theory decreased in this sequence: A_1_–RH (8.02 × 10^9^ M^−1^·s^−1^) > A_2_–RH (8.01 × 10^9^ M^−1^·s^−1^) > A_3_–RH (7.90 × 10^9^ M^−1^·s^−1^). Based on these results, the formation of A_1_−R^•^ was a kinetically favored process, while A_3_−R^•^ was thermodynamically preferred (Table 2 and Table 3). The stability of A_2_−R^•^ depended on the position of the OH substituent and the delocalization of unpaired electrons through the structure. No additional hydrogen bonds were observed.

Using the individual rate constants, the overall rate constant (*k*_overall_) was determined as a measure of the susceptibility of a compound to the AOPs involving HO^•^ (Table S2). All investigated compounds showed a high overall rate constant, with a negligible decrease in activity in the following order: A_1_–RH (1.21 × 10^10^ M^−1^·s^−1^) > A_2_–RH (1.19 × 10^10^ M^−1^·s^–1^) > A_3_–RH (1.18 × 10^10^ M^−1^·s^−1^). This correlated well with the experimental values obtained with EPR spectroscopy. This comparison proved the applicability of the QM-ORSA methodology for the prediction of the capacity and mechanisms of the radical action of the investigated compounds.

To evaluate the relative amounts of products, as well as the influence of individual reaction pathways on the overall capacity, branching ratios (*Г*_i_, %) were estimated (Table S2). The results in Table S2 indicate that HAT and SPLET were the dominant mechanisms in the HO^•^ removal process. Radicals in the HAT/PCET mechanism and anionic species in the SPL mechanism were formed in significant percentages: 16.06% (A_1_–RH), 15.78% (A_2_–RH), and 16.20% (A_3_–RH). Radicals formed during the electron transfer from the phenoxide anions to HO^•^ were present in the highest relative percentages: 67.40% (A_1_–RH), 66.16% (A_2_–RH), and 67.00% (A_3_–RH). The difference in the stabilization of anions and radicals overcame the importance of the substitution position in the cases of A_2_–RH and A_3_–RH, thus leading to the higher experimental and theoretical reactivity of the former towards HO^•^.

Finally, based on the *k*_overall_ value, it was possible to estimate the half-life (*τ*_1/2_) values of the investigated compounds exposed to HO^•^ radicals. The *τ*_1/2_ values calculated under physiological conditions and with different concentrations of HO^•^ are presented in Table S3. In natural water, [HO^•^]_aq_ concentrations ranged from 10^−18^ or 10^−14^ M to ca. 10^−10^ M in the AOP system. Specifically, at very low concentrations of HO^•^ in the range from approximately 10^−18^ to 10^−16^ M, the *τ*_1/2_ for degradation of A_1_–RH−A_3_–RH was in the range of 679.9–6.6 days. At HO^•^ concentrations between 10^−15^ and 10^−14^ M, the *τ*_1/2_ decreased to 16.8–1.6 h. At the HO^•^ concentration of 10^−10^ M, the half−life dropped to ~0.6 s. Thus, the effectiveness of AOP technology was demonstrated, as well as the importance of theoretical investigations and modeling of these processes.

### 3.5. Damage to a Target Biomolecule

**Figure 7:**
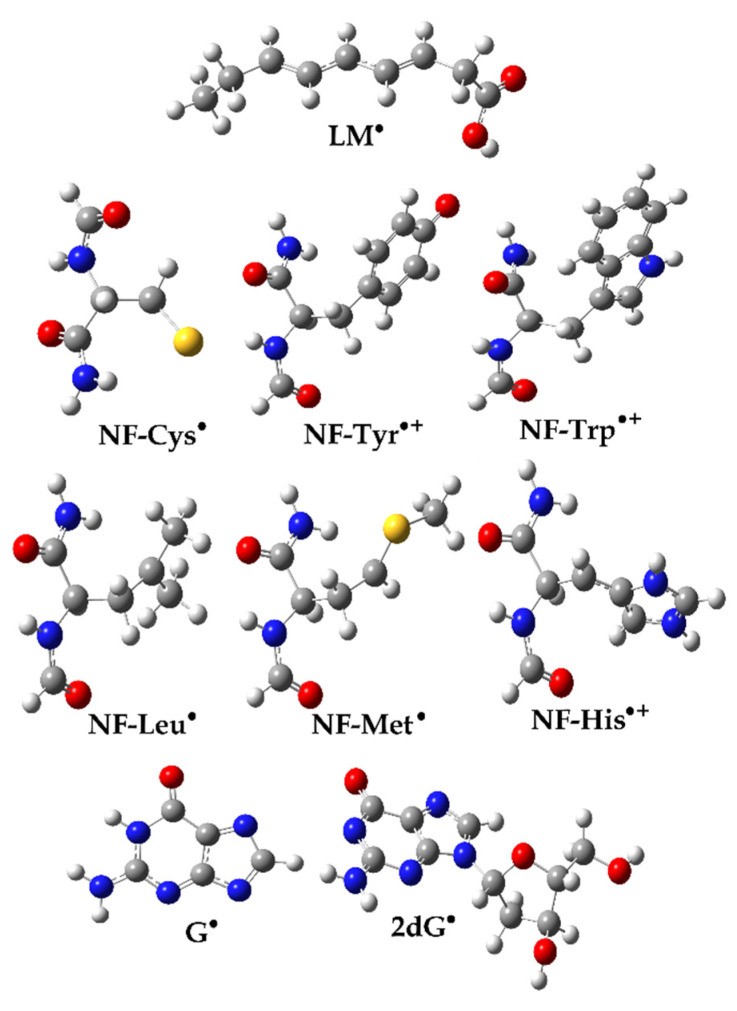
Optimized geometries of selected radicals/radical cations of biomolecular target compounds in water at M06-2X/6-311++G(d,p) level of theory.

To obtain information about the reactivity of the formed radical species (A_1_–O^•^, A_2_–O^•^, and A_3_–O^•^), interactions with the constituents of essential macromolecules—the phospholipid bilayer, proteins, and nucleic acids—were examined (Table 3). The optimized geometries of the reaction participants, neutral molecular targets, and corresponding radicals/radical cations are shown in Figure S8 and Figure 7. As expected, highly reactive radical species, such as HO^•^, interacted spontaneously with all investigated biomolecules (the lipid model (−190 kJ·mol^−1^), amino acid residues (<−111 kJ·mol^−1^), and nucleotides (<−97 kJ·mol^−1^)) except for NF-Trp (80 kJ·mol^−1^).

The lipid model (**LM**) is a model of linoleic acid that was employed on the basis of literature data [55]. This unsaturated fatty acid has two allylic H atoms that can react with radical species through the HAT mechanism [56]. Endergonic reactions between the formed radicals and **LM** (>62 kJ·mol^−1^) indicated that they do lead to the destruction of the building blocks of the cell membrane of living organisms.

Model compounds were chosen to represent the building blocks of proteins; amino acids have been successfully applied as reactive centers in the study of protein reactions [57]. Available literature data indicate that the selected amino acid residues (leucine (Leu), cysteine (Cys), methionine (Met), tyrosine (Tyr), histidine (His), and tryptophan (Trp)) are susceptible to radical attack [58]. These amino acid residues react with radical species through different mechanisms: SET (Tyr, Trp) and HAT (Cis, Leu, Met, His) [41]. Except for slightly exergonic Δ_r_*G* values with the amino acid residues NF-Leu (γ-site) (<−13 kJ·mol^−1^), NF-Met (γ-site) (<−10 kJ·mol^−1^), and NF-His (γ-site) (–2 kJ·mol^−1^), the newly formed radicals did not interact with the other investigated building blocks of the protein.

2′-Deoxyguanosine (2dG) and guanine (G), which is the most easily oxidized of all nucleobases, were chosen for oxidative DNA damage modeling [41,59]. The newly formed radical species did not interact with the building blocks of DNA molecules (nucleotides (>30 kJ·mol^−1^)), nor with the corresponding bases (>14 kJ·mol^−1^), as indicated by the endergonic Δ_r_*G* values.

### 3.6. Termination of A_n_–R^•^ Reactions by Synergistic Reactions with O_2_/NO and HO^•^

**Figure 8:**
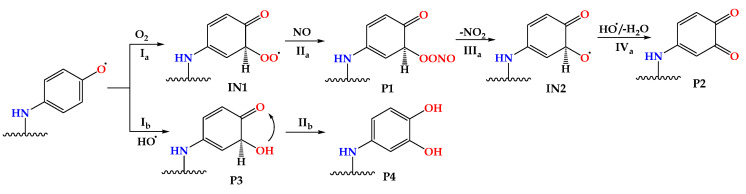
The proposed mechanism for the reaction between the formed radical species: A_n_–R^•^ and O_2_/NO (Ia, IIa, IIIa, IVa) and A_n_*–*R^•^ and HO^•^ (Ib, IIb).

Although the formed radicals did not show activity towards important constituents of biomolecules, the question of their further fate remained open. Based on available literature data [38], two possible reaction paths comprising radical inactivation and the formation of neutral products were proposed. For A_n_*–*R^•^ radical species, two carbon atoms located near the oxygen were chosen, and the hydrogen atom/proton abstraction process was modeled. The proposed reaction schemes for the mechanisms are presented in Figure 8, while the corresponding values of the thermodynamic parameters are summarized in Table 4.

**Figure 9:**
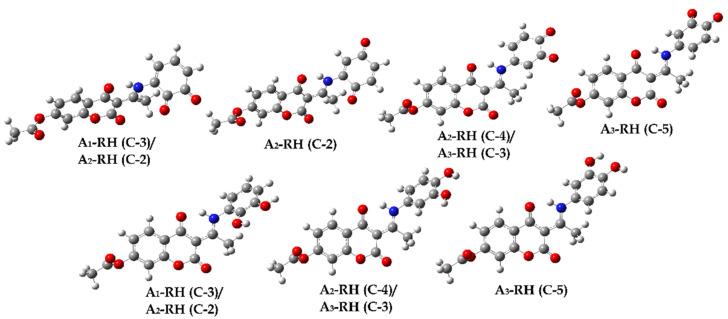
Optimized geometries of neutral final products P2 (**top**) and P4 (**bottom**) in water at M06−2X/6−311++G(d,p) level of theory.

Available literature data indicate the ability of radicals to react synergistically with O_2_/NO and form neutral products (Figure 8 and Figure 9, P2) [38]. A variable concentration of dissolved molecular oxygen (O_2_) in water enables the reaction with radical species leading to the formation of a corresponding peroxide adduct (IN1, Ia, Figure S9), which can further react with the NO present in natural and wastewater to form a radical adduct (P1, IIa, Figure S10). The next step involved the spontaneous intramolecular separation of NO_2_ molecules (IIIa) with the formation of the intermediate radical adduct IN2 (Figure S11). In this specific context, Δ_r_*G* was exergonic and thermodynamically favored, especially in the case of the addition of molecular oxygen (Table 4). Finally, the action of HO^•^, through a highly exergonic mechanism (<−414 kJ·mol^−1^) that has already been observed in our previous research, was found to result in the intramolecular separation of water molecules (IVa) and the formation of a neutral product (P2, Figure 9).

Another mechanism has also been postulated and discussed in detail in previous work focused on AOP systems [24,24,53]. It involves the reaction of a formed radical with another HO^•^ radical (Ib), resulting in the formation of a neutral product (P3, Figure S12). In the next step, through keto–enol tautomerism (IIb) in a highly exergonic process (>−101 kJ·mol^−1^, Table 2), a catechol-type product is formed (P4).

### 3.7. Ecotoxicological Approach

The resulting stable oxidation products (P2, P4) were subjected to acute and chronic ecotoxicity estimation investigations for aquatic organisms: fish, daphnia, and green algae. The results, obtained as part of the ECOSAR program, together with the reference values published by the European Union (acute toxicity, described in Annex VI of Directive 67/548/EEC) [60] and in the Chinese hazard evaluation guidelines for new chemical substances (chronic toxicity, HJ/T 154-2004) [61], are summarized in Table 5.

All starting compounds (A_1_–RH, A_2_–RH, and A_3_–RH) showed harmful acute (<15.10 mg·L^−1^) and chronic toxic effects (<1 mg·L^−1^) on fish. On the other hand, all oxidation products showed less acute harmful (>18.70 mg·L^−1^) and chronic (>1.21 mg·L^−1^) toxicity. In contrast, the oxidative product P3 of all compounds was entirely harmless in terms of acute (>157.00 mg·L^−1^) and chronic toxicity (>11.10 mg·L^−1^). In general, oxidation product P4 showed a less acute harmful effect on daphnia (>36.10 mg·L^−1^) in comparison to neutral compounds, while being utterly harmless in terms of chronic toxicity (>20.30 mg·L^−1^). Oxidation products P3 and P4 showed fewer harmful effects in terms of acute (>13.10 mg·L^−1^) and chronic toxicity (>4.43 mg·L^−1^) in comparison to the neutral starting compounds. A similar trend was observed in the interpretation of the ecotoxicological status of green algae.

## 4. Conclusions

Application of advanced oxidation processes to stable coumarin derivatives is one way to remove them from wastewater and decrease the toxicity towards aquatic organisms. Three aminophenol derivatives of 4,7–hydroxycoumarin and HO^•^ were employed for experimental and theoretical examination of the relevant reaction mechanisms. Based on the results of EPR measurements, it can be concluded that the investigated compounds showed high reactivity towards the HO^•^ radical, with a decrease in reactivity in the following order A_1_–RH (91%) > A_2_–RH (88%) > A_3_–RH (81%). At the physiological pH value, all three compounds presented as neutral species (>99%), followed by the monoanionic forms. Analysis of the thermodynamic and kinetic parameters confirmed that the hydrogen atom transfer (HAT), sequential proton loss followed by electron transfer (SPLET), and radical adduct formation (RAF) mechanisms were the operative reaction pathways in the degradation of the studied compounds induced by the HO^•^ radical. The hydrogen atom and electron transfers represented diffusion-controlled reactions, while the RAF rate constants were between 10^3^ and 10^7^ M^−1^s^−1^, depending on the reaction site. Estimated overall rate constants (*k*_overall_) decreased slightly in the order: A_1_–RH (1.21 × 10^10^ M^−1^·s^−1^) > A_2_–RH (1.19 × 10^10^ M^−1^·s^−1^) > A_3_–RH (1.18 × 10^10^ M^−1^·s^−1^), as obtained using the QM−ORSA methodology, showing excellent agreement with the reactivity order observed using EPR spectroscopy. The anionic species present greatly influenced the overall rate constant, leading to the obtained order of reactivity. The stability of the formed radical and anionic species resulted from extended delocalization and weak interactions between groups within compounds. The estimated values of the branching ratios (*Г_i_*, %) of products indicated that the degradation of the investigated compounds induced by HO^•^ mainly occurred via HAT and SPLET mechanisms, with half-life (*τ*_1/2_) values of ca. 0.6 s. The distinctly endergonic Δ_r_*G* values for the reaction of the formed radicals (A_1_−R^•^, A_2_−R^•^, and A_3_−R^•^) and the biomolecule building blocks (linoleic acid, amino acids, and guanine) demonstrated the importance of the lower toxicity of products in AOPs. As expected, the Δ_r_*G* values were more endergonic compared to the interaction of the HO^•^ radical and the investigated macromolecular targets. The formed radical species could further interact with the O_2_/NO and HO^•^ present in wastewaters to end the cycle and form neutral species. The thermodynamic favorability of these reaction pathways was reflected in the highly exergonic Δ_r_*G* values for the four/two steps of the proposed mechanisms The formed neutral products had lower acute and chronic toxicity than the starting neutral compounds, as estimated in the ECOSAR program, towards daphnia, fish, and green algae. The presented experimental/theoretical results demonstrate the applicability of the proposed mechanisms. They open the way for future analysis and application of the mentioned theoretical approach in developing advanced oxidation processes and highlight the need for reliable determination of toxicity towards aquatic organisms.

## Figures and Tables

**Table 1:** Estimated values of reaction Gibbs energies (Δ_r_*G*) in kJ·mol^−1^ for reactions between coumarin derivatives (A_1_–RH, A_2_–RH, A_3_–RH) and HO^•^ at 298.15 K.

**Species**	**HO^•^**				
	**HAT/PCET**	**SET−PT**		**SPLET**	
	**Δ_r_*G*_HAT/PCET_**	**Δ_r_*G*_SET_**	**Δ_r_*G*_PT_**	**Δ_r_*G*_SPL_**	**Δ_r_*G*_ET_**
A_1_–RH	−127	139	−266	−115	−13
A_2_–RH	−124	144	−268	−103	−21
A_3_–RH	−126	122	−248	−115	−28
**Positions**	**RAF,** Δ_r_*G*_RAF_				
	**A_1_–RH**	**A_2_–RH**		**A_3_–RH**	
C3	−7	−15		−5	
C5	−41	−43		−37	
C6	−18	−21		−17	
C7	−44	−43		−40	
C8	−34	−36		−35	
C9	−31	−35		−29	
C10	2	−4		4	
C1′	−27	−27		−24	
C1″	−35	−27		−34	
C2″	−43	−38		−17	
C3″	−30	−34		−31	
C4″	−22	−42		−40	
C5″	−24	−22		−32	
C6″	−31	−21		−30	

**Table 2:** Estimated values of kinetic parameters at 298.15 K: activation Gibbs energy (Δ*G*_a_, kJ·mol^−1^) and rate constants of the bimolecular chemical reactions (M^−1^ s^−1^) between the investigated compounds A_1_–RH, A_2_–RH, and A_3_–RH and HO^•^ estimated with the Eckart method *(k*_ZCT_0_). The *k*^ET^ values (M^−1^ s^−1^) represent rate constants calculated with Marcus theory.

Species	HAT/PCET		SPLET			
	$\Delta G_{a}^{HAT/PCET}$	$k_{ZCT\_0}^{HAT/PCET}$	$\Delta G_{a}^{SPL}$	$k^{SPL}$	$\Delta G_{a}^{ET}$	$k^{ET}$
A_1_–RH	~0	1.91 × 10^9^	~0	1.91 × 10^9^	2	8.02 × 10^9^
A_2_–RH					2	8.01 × 10^9^
A_3_–RH					6	7.90 × 10^9^
**Position**	**A_1_** **–RH**		**A_2_** **–RH**		**A_3_** **–RH**	
	** $\Delta G_{a}^{RAF}$ **	** $k_{ZCT\_0}^{RAF}$ **	** $\Delta G_{a}^{RAF}$ **	** $k_{ZCT\_0}^{RAF}$ **	** $\Delta G_{a}^{RAF}$ **	** $k_{ZCT\_0}^{RAF}$ **
C3	40	1.61 × 10^7^	41	1.48 × 10^7^	47	1.22 × 10^6^
C5	54	7.15 × 10^4^	51	2.77 × 10^5^	53	1.11 × 10^5^
C6	56	3.34 × 10^4^	53	1.35 × 10^5^	55	4.66 × 10^4^
C7	60	7.54 × 10^3^	51	2.80 × 10^5^	63	2.31 × 10^3^
C8	47	1.33 × 10^6^	44	3.46 × 10^6^	53	1.19 × 10^5^
C9	56	3.12 × 10^4^	53	1.35 × 10^5^	57	2.12 × 10^4^
C10	50	4.15 × 10^5^	46	1.85 × 10^6^	52	1.49 × 10^5^
C1′	54	8.03 × 10^4^	50	3.72 × 10^5^	55	4.55 × 10^4^
C1″	41	1.29 × 10^7^	51	2.53 × 10^5^	41	1.34 × 10^7^
C2″	47	1.35 × 10^6^	36	4.46 × 10^7^	49	5.96 × 10^5^
C3″	41	1.20 × 10^7^	48	1.48 × 10^7^	39	2.44 × 10^7^
C4″	48	7.15 × 10^5^	33	1.39 × 10^8^	52	1.46 × 10^5^
C5″	42	7.94 × 10^6^	51	2.78 × 10^5^	39	2.55 × 10^7^
C6″	42	3.34 × 10^6^	32	5.66 × 10^7^	43	5.55 × 10^6^

**Table 3:** Estimated values of reaction Gibbs energies (Δ_r_*G*) in kJ·mol^−1^ for reactions between the biologically important target molecules and the formed A_1_−R^•^, A_2_−R^•^, A_3_−R^•^ radicals at 298.15 K.

Target Molecule	A_1_–O^•^	A_2_–O^•^	A_3_–O^•^	HO^•^
LM	62	65	63	−190
NF−Leu (γ−site)	−16	−13	−15	−111
NF−Cys (SH site)	18	21	19	−145
NF−Tyr (OH site)	9	12	10	−136
NF−Tyr (SET)	139	148	155	127
NF−Trp (SET)	92	101	108	80
NF−Met (γ−site)	−13	−10	−12	−114
NF−His (SET)	−5	−2	−3	−123
G	17	14	16	−110
2dG	30	27	29	−97

**Table 4:** Estimated values of reaction Gibbs energies (Δ_r_*G*) in kJ·mol^−1^ for reactions between the formed radicals A_1_*−*R^•^, A_2_*−*R^•^, and A_3_*−*R^•^ and O_2_/NO in different sequential reaction pathways (I_a_, II_a_, III_a_, IV_a_) or HO^•^ (I_b_, II_b_) at 298.15 K.

Radical	Position	I_a_	II_a_	III_a_	IV_a_	I_b_	II_b_
A_1_*–*O^•^	C*-*3	−98	−32	−15	−416	−207	−101
	C*-*5	−112	−28	−10	−421	−207	/
A_2_*–*O^•^	C*−*2	−110	−21	−29	−419	−211	−107
	C*-*4	−116	−21	−24	−414	−214	−107
A_3_*–*O^•^	C*-*3	−95	−31	−9	−438	−200	−116
	C*-*5	−98	−28	−27	−421	−211	−111

**Table 5:** Estimated ecotoxicity values of A_1_–RH, A_2_–RH, and A_3_–RH and their oxidation products in relation to aquatic organisms (mg·L^−1^) at pH = 7.4.

Products	Acute Toxicity *			Chronic Toxicity *		
	Fish LC_50_	Daphnia LC_50_	Green Algae EC_50_	Fish ChV	Daphnia ChV	Green Algae ChV
A_1_–RH	9.40	17.20	6.04	0.60	8.31	2.29
P1-C3/C5	27.40	3.54	2.49	1.24	0.32	0.88
P2-C3	99.6	11.60	10.1	6.28	0.93	3.28
P2-C5	59.1	7.40	5.73	3.30	0.60	1.92
P3-C3	49.8	6.10	4.77	2.67	0.51	1.61
P3-C5	199.00	22.00	21.20	14.80	1.67	6.65
P4-C3	18.7	36.10	13.70	1.21	20.30	4.43
A_2_–RH	15.10	28.60	10.60	0.95	15.30	3.60
P1-C2/C4	86.70	10.30	8.58	5.15	0.84	2.83
P2-C2/C4	99.60	11.60	10.10	6.28	0.93	3.28
P3-C2/C4	157.00	17.60	16.40	11.10	1.37	5.22
P4-C2	18.70	36.10	13.70	1.21	20.30	4.43
P4-C4	30.00	60.00	24.10	2.10	36.90	6.69
A_3_–RH	15.10	28.60	10.60	0.95	15.30	3.60
P1-C3/C5	86.70	10.30	8.58	5.15	0.84	2.83
P2-C3/C5	99.6	11.60	10.10	6.28	0.93	3.28
P3-C3/C5	157.00	17.60	16.40	11.10	1.37	5.22
P4-C3/C5	30.00	60.00	24.10	2.10	36.90	6.69

* Reference values [60,61]. Not harmful: log LC_50_ > 100 or log EC_50_ > 100. Log ChV > 10. Harmful: 10 < log LC_50_ < 100 or 10 < log EC_50_ < 100. 1 < log ChV < 10. Toxic: 1 < log LC_50_ < 10 or 1 < log EC_50_ < 10. 0.1 < log ChV < 1. Very toxic: log LC_50_ < 1 or log EC_50_ < 1. Log ChV < −0.1.

## Data Availability

Data are contained within the article and Supplementary Materials.

## Supplementary Materials

The following are available online at https://www.mdpi.com/article/10.3390/ijerph20032046/s1, Figure S1: Optimized geometries of radical adducts formed between A_1_–RH and HO^•^ at M06-2X/6-311++G(d,p) level of theory with characteristic bond distances (Å); Figure S2: Optimized geometries of radical adducts formed between A_2_–RH and HO^•^ at M06-2X/6-311++G(d,p) level of theory with characteristic bond distances (Å); Figure S3: Optimized geometries of radical adducts formed between A_3_–RH and HO^•^ at M06-2X/6-311++G(d,p) level of theory with characteristic bond distances (Å); Table S1. Estimated values of kinetic parameters: activation energy (Δ*G*_a_, kJ·mol^−1^) and rate constants of the bimolecular chemical reactions (M^−1^·s^–1^) between the investigated compounds A_1_–RH, A_2_–RH, and A_3_–RH and HO^•^ estimated using conventional transition state theory (*k*_TST_); Figure S4: Dependence of total energy (a.u.) on the characteristic HO–H2 (A_1_–RH, top), HO–H3 (A_2_–RH, center), and HO–H4 (A_3_–RH, bottom) distances (Å) for the HAT/PCET mechanism; Figure S5: Optimized transition state geometries for the formation of radical adducts between A_2_–RH and HO^•^ in water at M06-2X/6-311++G(d,p) level of theory; Figure S6: Optimized transition state geometries for the formation of radical adducts between A_3_–RH and HO^•^ in water at M06-2X/6-311++G(d,p) level of theory; Figure S7: Dependence of total energy (a.u.) on the characteristic HO–H2 (A_1_–RH, top), HO–H3 (A_2_–RH, center), and HO–H4 (A_3_–RH, bottom) distances (Å) for the SPL mechanism; Table S2. Estimated overall rate constants (*k*_overall_) and branching ratios (*Г_i_*, %) at pH = 7.4 for newly synthesized coumarin derivatives A_1_–RH–A_3_–RH; Table S3. Half−lives (*τ*_1/2_) of investigated compounds (A_1_–RH–A_3_–RH) at physiological pH (7.4) and different concentrations (M) of HO^•^ radicals; Figure S8: Optimized geometries of the selected biomolecular target compound in water at M06-2X/6-311++G(d,p) level of theory; Figure S9. Optimized geometries of intermediate radical adducts (IN1) formed in the reactions between A_1_–RH (top), A_2_–RH (center), and A_3_–RH (bottom) and O_2_ in water at M06-2X/6-311++G(d,p) level of theory; Figure S10. Optimized geometries of intermediate adducts (P1) formed in the reactions between A_1_–R^•^ (IN1, top), A_2_–R^•^ (IN1, center), and A_3_–R^•^ (IN1, bottom) and NO in water at M06-2X/6-311++G(d,p) level of theory; Figure S11. Optimized geometries of intermediate radical adducts (IN2) formed in the intramolecular separation of NO_2_ molecules in A_1_–R^•^ (P1, top), A_2_–R^•^ (P1, center), and A_3_–R^•^ (P1, bottom) in water at M06-2X/6-311++G(d,p) level of theory; Figure S12. Optimized geometries of intermediate adducts (P3) formed in the reactions between A_1_–R^•^ (top), A_2_–R^•^ (center), and A_3_–R^•^ (bottom) and HO^•^ in water at M06-2X/6-311++G(d,p) level of theory.
