# Supplementary material for: Degradation Mechanisms of 4,7-Dihydroxycoumarin Derivatives in Advanced Oxidation Processes: Experimental and Kinetic DFT Study
Source: Int J Environ Res Public Health. 2023 Jan 22;20(3):2046. doi: 10.3390/ijerph20032046 (PMC9916318; doi:10.3390/ijerph20032046)
Supplement: Supplementary file 1 [file ijerph-20-02046-s001.zip › ijerph-2120462-supplementary.pdf]

Supplementary Material

for

Degradation Mechanism of Previously Synthesized 4,7-Dihydroxycoumarin Derivatives in Advanced Oxidation Process-Experimental and Kinetic DFT Study

Žiko Milanović [1], Dušan Dimić [2,*], Erik Klein [3], Monika Biela [3], Vladimír Lukeš [3], Milan Žižić [4], Edina Avdović [1], Drago Bešlo [5], Radiša Vojinović [6], Jasmina Dimitrić Marković [2] and Zoran Marković [2,*]

[1] *Department of Science, Institute for Information Technologies, University of Kragujevac, Jovana Cvijića bb, 34000 Kragujevac, Serbia*

[2] *Faculty of Physical Chemistry, University of Belgrade, 12-16 Studentski trg, 11000 Belgrade, Serbia*

[3] *Institute of Physical Chemistry and Chemical Physics, Slovak University of Technology in Bratislava, Radlinského 9, SK-812 37 Bratislava, Slovakia*

[4] *Life Sciences Department, Institute for Multidisciplinary Research, University of Belgrade, Kneza Višeslava 1, 11030 Belgrade, Serbia*

[5] *Department of Agroecology and Environmental Protection, Faculty of Agrobiotechnical Sciences Osijek, University Josip Juraj Strossmayer Osijek, Vladimir Prelog 1, Osijek, Croatia*

[6] *Faculty of Medical Sciences, University of Kragujevac, Svetozara Markovića 69, 34000 Kragujevc, Re-public of Serbia*

*** Correspondence:** ddimic@ffh.bg.ac.rs (D.D.); zmarkovic@uni.kg.ac.rs (Z.M.); Tel.: +381-34-610-01-95 (Z.M.)

Table of Contents:

Figure S1. Optimized geometries of formed radical adducts between **A_1-RH** and $HO^•$ at M06-2X/6-311++G(d,p) level of theory with characteristic bond distances (Å)……………………**.3**

Figure S2. Optimized geometries of formed radical adducts between **A_2-RH** and $HO^•$ at M06-2X/6-311++G(d,p) level of theory with characteristic bond distance (Å)………………………**4**

Figure S3. Optimized geometries of formed radical adducts between **A_3-RH** and $HO^•$ at M06-2X/6-311++G(d,p) level of theory with characteristic bond distance (Å)………………………**5**

Table S1. Estimated values of kinetic parameters: activation energy (ΔG_a, kJ mol^{-1}), rate constants of the bimolecular chemical reaction (M^{-1}s^{-1}) between the investigated compounds **A_1-RH**, **A_2-RH**, **A_3-RH**, and $HO^•$ estimated by the conventional transition state theory (k_{TST})…………………………..**6**

Figure S4. Dependence of total energy (a.u.) on the characteristic HO–H2 (**A_1-RH,** up) HO–H3 (**A_2-RH**, medium) and HO–H4 (**A_4-RH**, down) distance (Å) for the HAT/PCET mechanism………..**7**

Figure S5. Optimized transition state geometries for the formation of radical adducts between **A_2-RH** and $HO^•$ in water at M06-2X/6-311++G(d,p) level of theory……………………………………..**8**

Figure S6. Optimized transition state geometries for the formation of radical adducts between **A_3-RH** and $HO^•$ in water at M06-2X/6-311++G(d,p) level of theory……………………………………..**9**

Figure S7. Dependence of total energy (a.u.) on the characteristic HO–H2 (**A_1-RH,** up) HO–H3 (**A_2-RH**, medium) and HO–H4 (**A_3-RH**, down) distance (Å) for the SPL mechanism………………...**10**

Table S2. Estimated overall rate constants ($k_{overall}$) and branching ratios (Γ_i,%) at pH=7.4 for newly synthesized coumarin derivatives **A_1-RH – A_3-RH**…………………………………………...**11**

Table S3. Half-life ($\tau_{1/2}$) of investigated compounds (**A_1-RH – A_3-RH**) at physiological pH (7.4) and different concentrations (M) of $HO^•$ radical……………………………………………………..**11**

Figure S8. Optimized geometries of selected biomolecular target compounds in water at M06-2X/6-311++G(d,p) level of theory…………………………………………………………………**12**

Figure S9. Optimized geometries of intermediate radical adducts, **IN1**, formed in the reaction **A_1-RH** (up), **A_2-RH** (medium), **A_3-RH** (down) and O_2 in water at M06-2X/6-311++G(d,p) level of theory……………………………………………………………………………………**13**

Figure S10. Optimized geometries of intermediate adducts, **P1**, formed in the reaction **A_1-R$^•$** (IN1, up), **A_2-R$^•$** (IN1, medium), **A_3-R$^•$** (IN1, down) and NO in water at M06-2X/6-311++G(d,p) level of theory……………………………………………………………………………………**14**

Figure S11. Optimized geometries of intermediate radical adducts, **IN2**, formed in the reaction intramolecular separation NO_2 molecules of **A_1-R$^•$** (P1, up), **A_2-R$^•$** (P1, medium), **A_3-R$^•$** (P1, down) in water at M06-2X/6-311++G(d,p) level of theory…………………………………………………**15**

Figure S12. Optimized geometries of intermediate adducts, **P3**, formed in the reaction **A_1-R$^•$** (up), **A_2-R$^•$** (medium), **A_3-R$^•$** (down) and $HO^•$ in water at M06-2X/6-311++G(d,p) level of theory………**16**

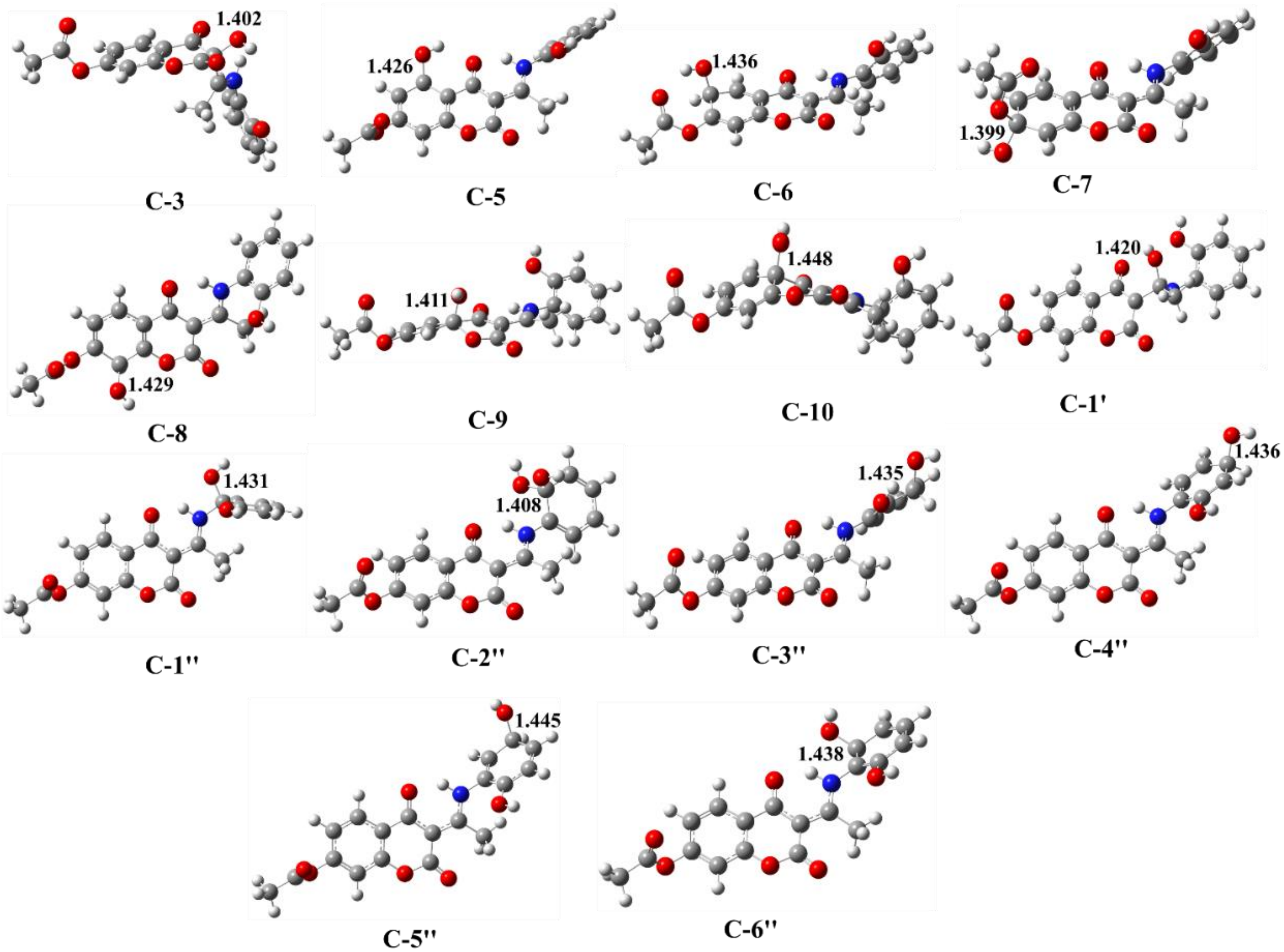

Figure S1. Optimized geometries of formed radical adducts between **A_1-RH** and $HO^{\bullet}$ at M06-2X/6-311++G(d,p) level of theory with characteristic bond distances (Å)

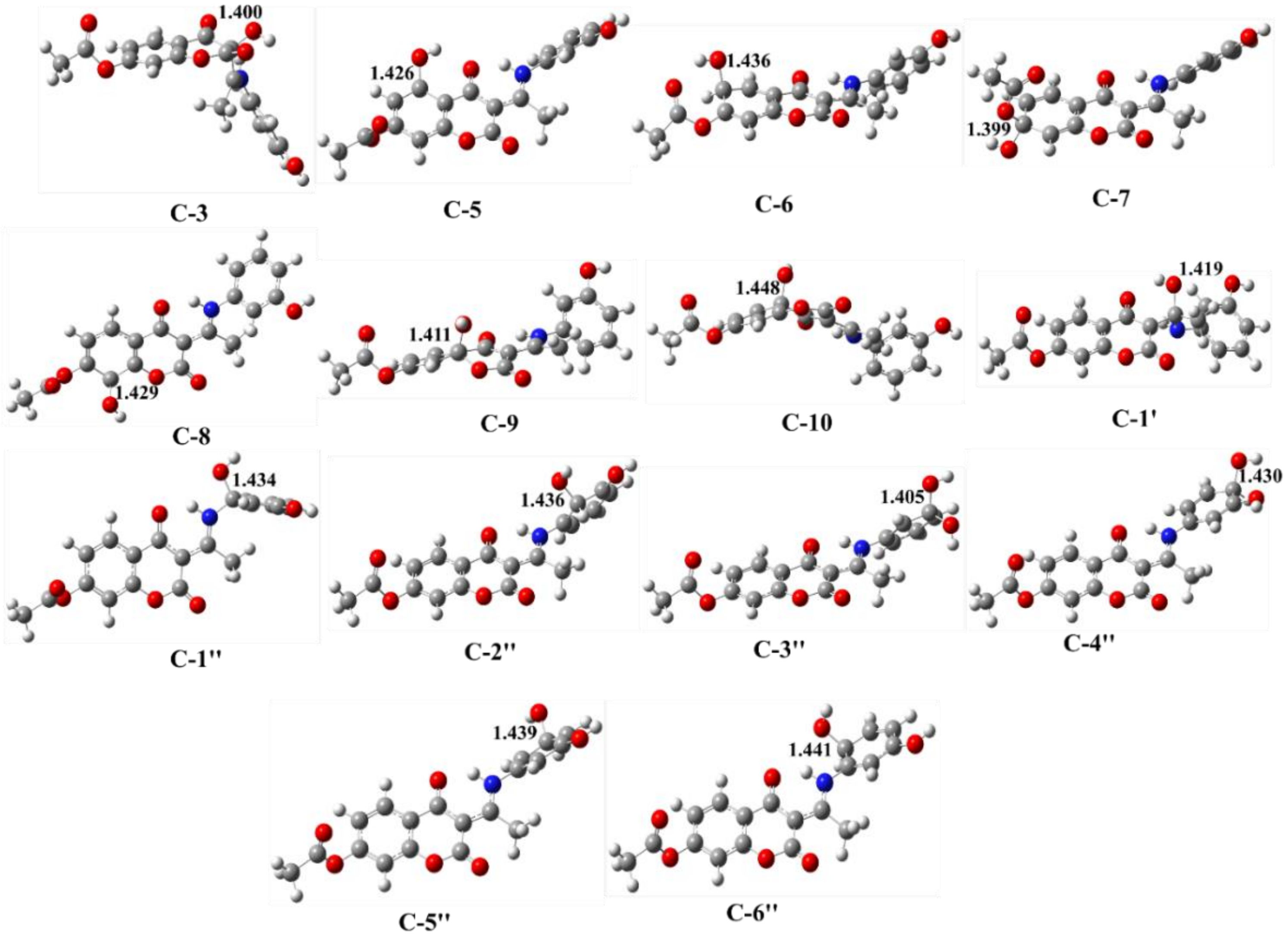

Figure S2. Optimized geometries of formed radical adducts between **A$_2$-RH** and HO$^{\bullet}$ at M06-2X/6-311++G(d,p) level of theory with characteristic bond distance (Å)

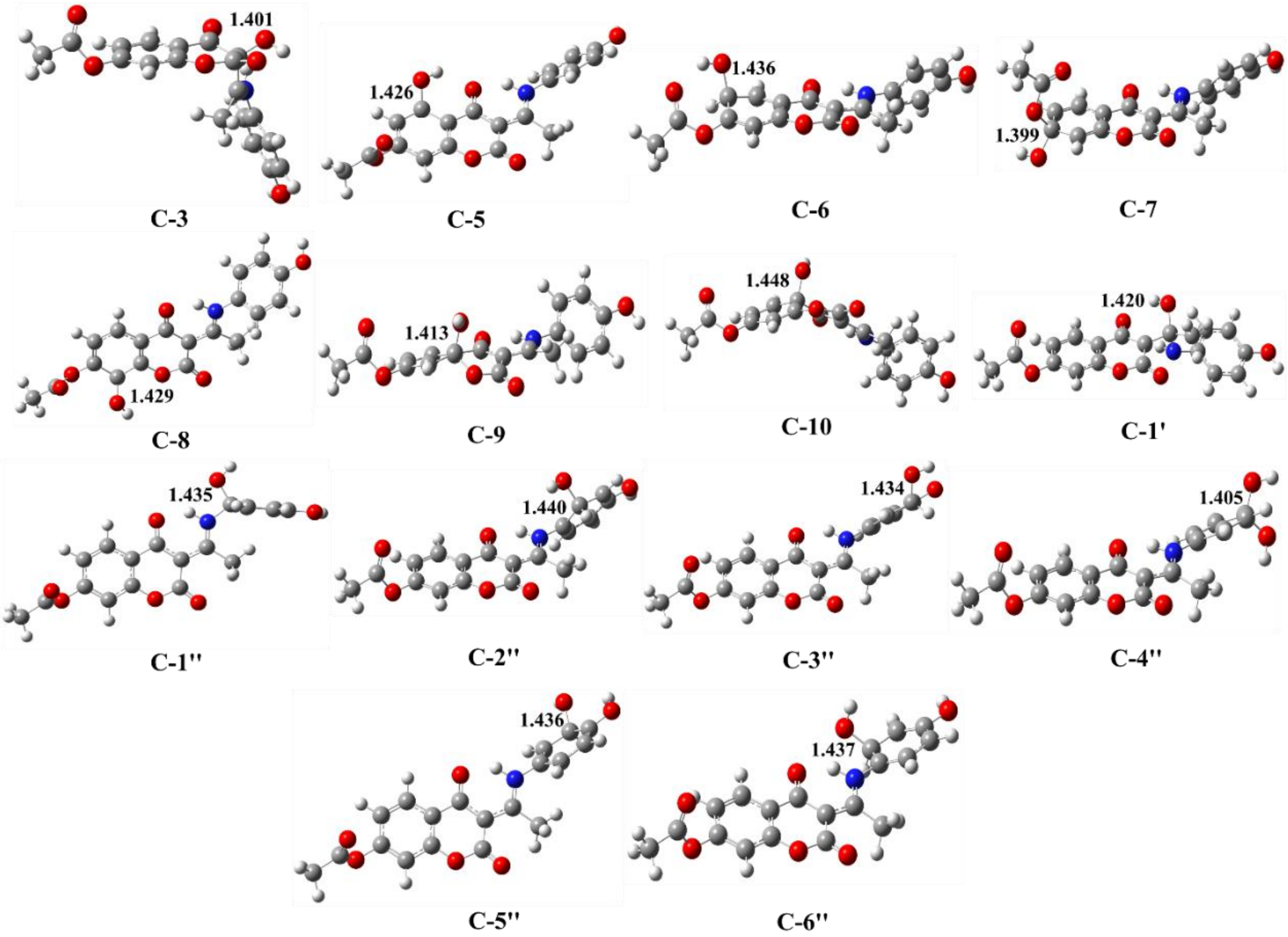

Figure S3. Optimized geometries of formed radical adducts between **A_3-RH** and HO$^{\bullet}$ at M06-2X/6-311++G(d,p) level of theory with characteristic bond distance (Å)

Table S1. Estimated values of kinetic parameters: activation energy (ΔG_a, kJ mol^{-1}), rate constants of the bimolecular chemical reaction (M^{-1}s^{-1}) between the investigated compounds $\mathbf{A_1}$**-RH**, $\mathbf{A_2}$**-RH**, $\mathbf{A_3}$**-RH**, and HO$^{\bullet}$ estimated by the conventional transition state theory (k_{TST})

RAF						
Position	$\mathbf{A_1}$**-RH**		$\mathbf{A_2}$**-RH**		$\mathbf{A_3}$**-RH**	
	ΔG_a^{RAF}	k_{TST}^{RAF}	ΔG_a^{RAF}	k_{TST}^{RAF}	ΔG_a^{RAF}	k_{TST}^{RAF}
**C-3**	40	$1{,}26\times10^{7}$	41	$1{,}16\times10^{7}$	47	$9{,}40\times10^{5}$
**C-5**	54	$4{,}53\times10^{4}$	51	$1{,}76\times10^{5}$	53	$7{,}02\times10^{4}$
**C-6**	56	$2{,}27\times10^{4}$	53	$9{,}17\times10^{4}$	55	$3{,}15\times10^{4}$
**C-7**	60	$4{,}96\times10^{3}$	51	$1{,}85\times10^{5}$	63	$1{,}53\times10^{3}$
**C-8**	47	$1{,}01\times10^{6}$	44	$2{,}63\times10^{6}$	53	$8{,}98\times10^{4}$
**C-9**	56	$2{,}00\times10^{4}$	53	$8{,}71\times10^{4}$	57	$1{,}37\times10^{4}$
**C-10**	50	$3{,}16\times10^{5}$	46	$1{,}41\times10^{6}$	52	$1{,}13\times10^{5}$
**C-1′**	54	$6{,}35\times10^{4}$	50	$2{,}95\times10^{5}$	55	$3{,}63\times10^{4}$
**C-1″**	41	$1{,}08\times10^{7}$	51	$1{,}80\times10^{5}$	41	$1{,}18\times10^{7}$
**C-2″**	47	$1{,}02\times10^{6}$	36	$6{,}26\times10^{7}$	49	$4{,}26\times10^{5}$
**C-3″**	41	$9{,}68\times10^{6}$	48	$1{,}16\times10^{7}$	39	$2{,}02\times10^{7}$
**C-4″**	48	$5{,}03\times10^{5}$	33	$2{,}83\times10^{8}$	52	$1{,}10\times10^{5}$
**C-5″**	42	$6{,}25\times10^{6}$	51	$1{,}76\times10^{5}$	39	$2{,}12\times10^{7}$
**C-6″**	42	$2{,}27\times10^{4}$	32	$4{,}37\times10^{8}$	43	$4{,}16\times10^{6}$

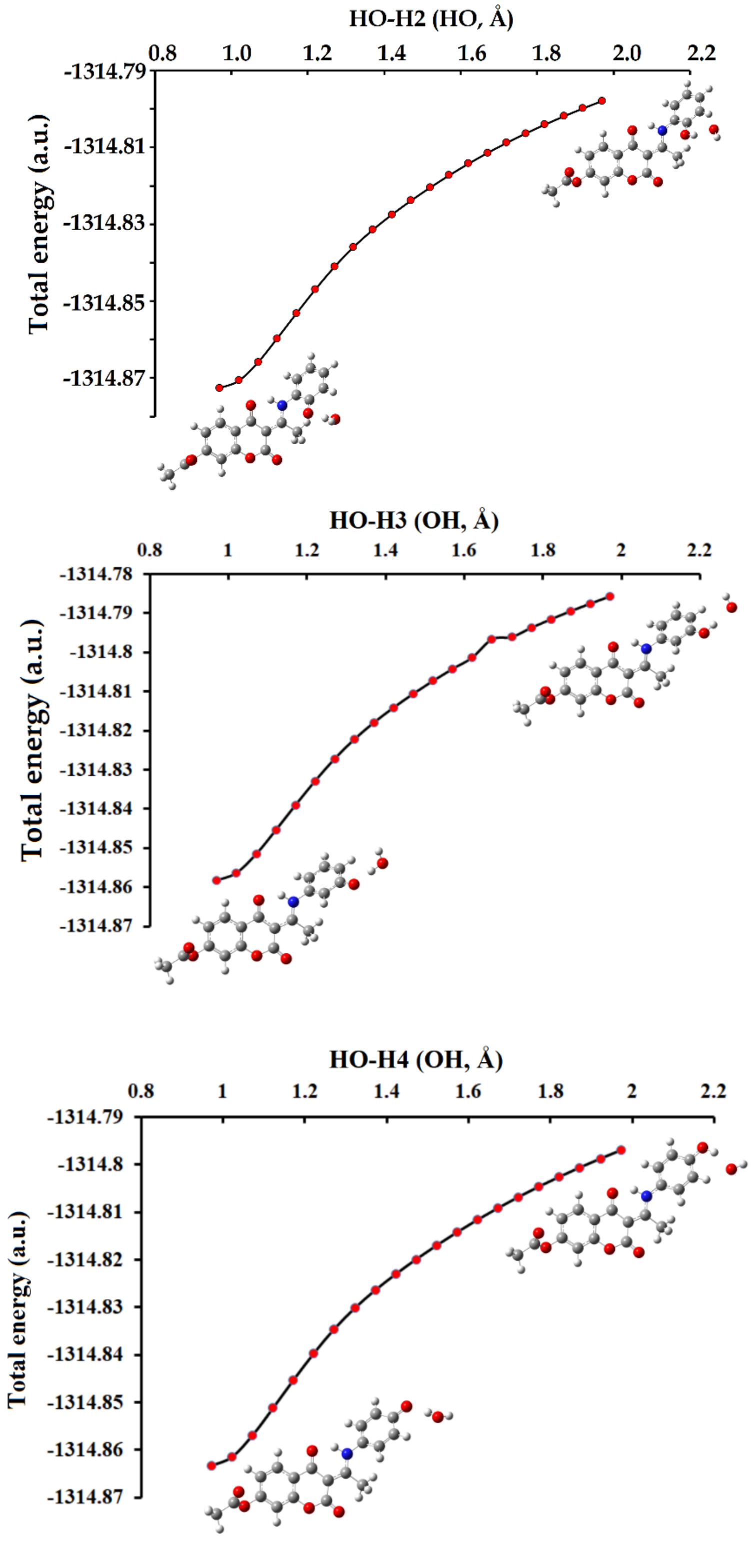

Figure S4. Dependence of total energy (a.u.) on the characteristic HO–H2 (**A$_1$-RH,** up) HO–H3 (**A$_2$-RH**, medium) and HO–H4 (**A$_4$-RH**, down) distance (Å) for the HAT/PCET mechanism

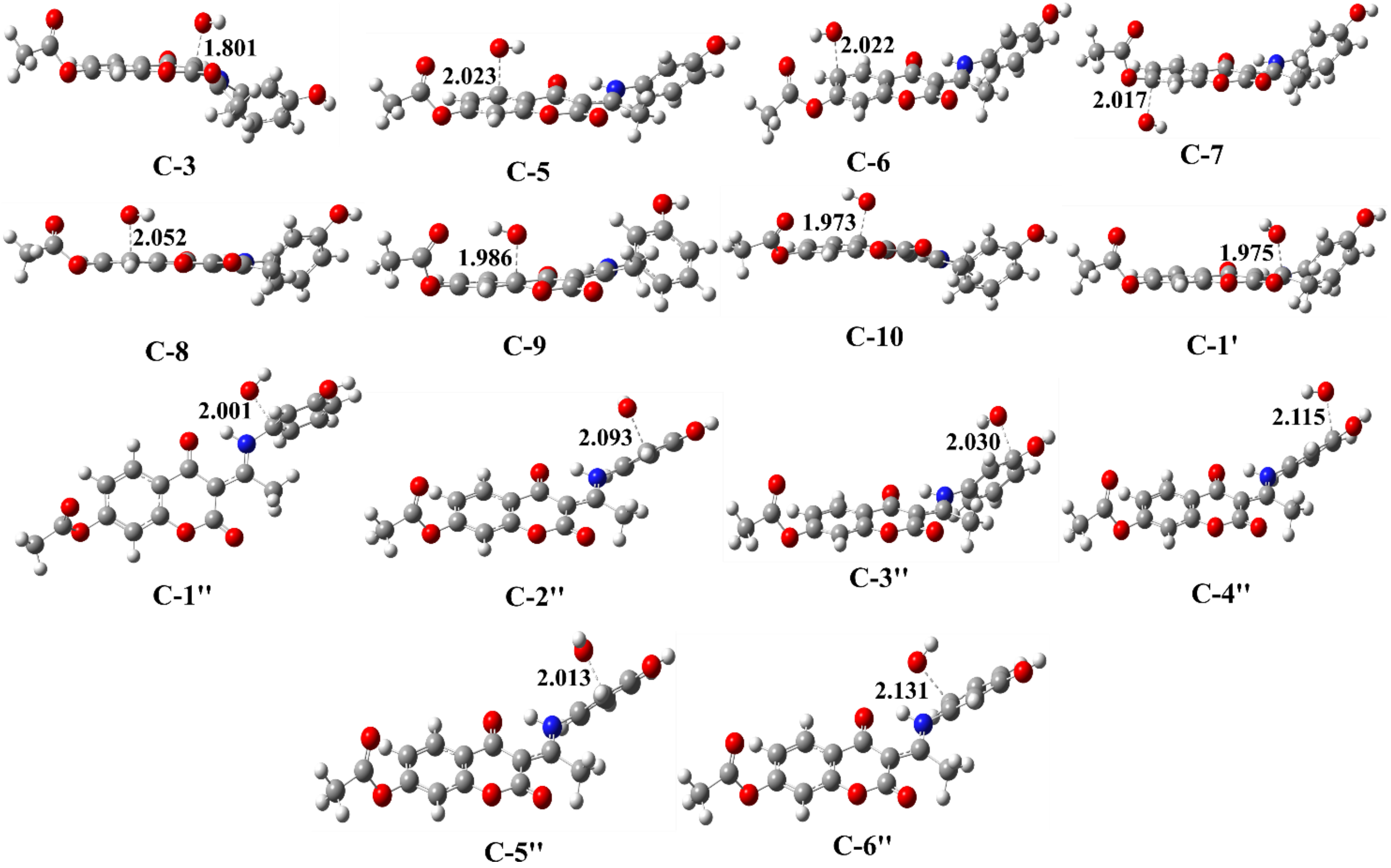

Figure S5. Optimized transition state geometries for the formation of radical adducts between **A_2-RH** and HO• in water at M06-2X/6-311++G(d,p) level of theory

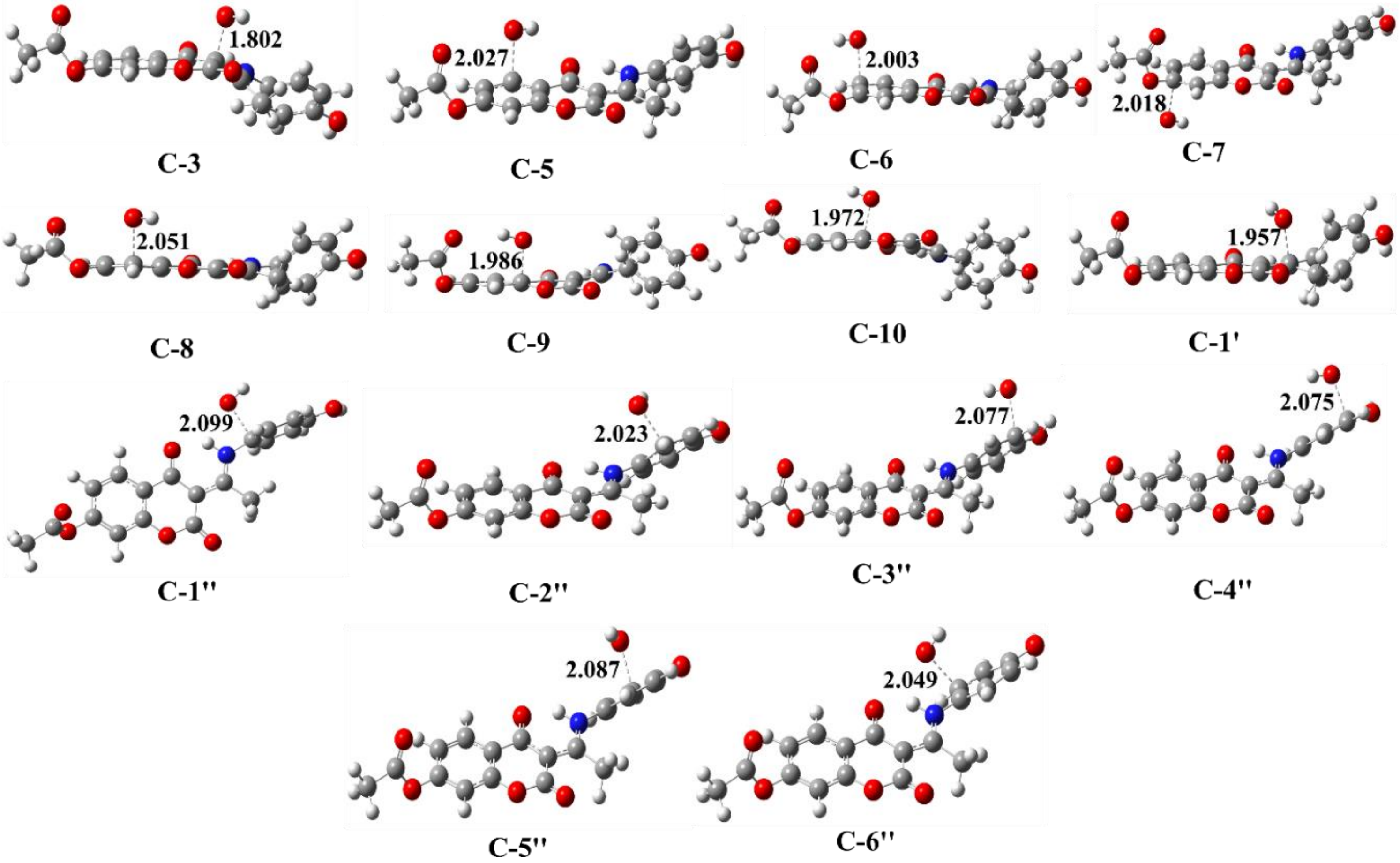

Figure S6. Optimized transition state geometries for the formation of radical adducts between **A₃-RH** and HO• in water at M06-2X/6-311++G(d,p) level of theory

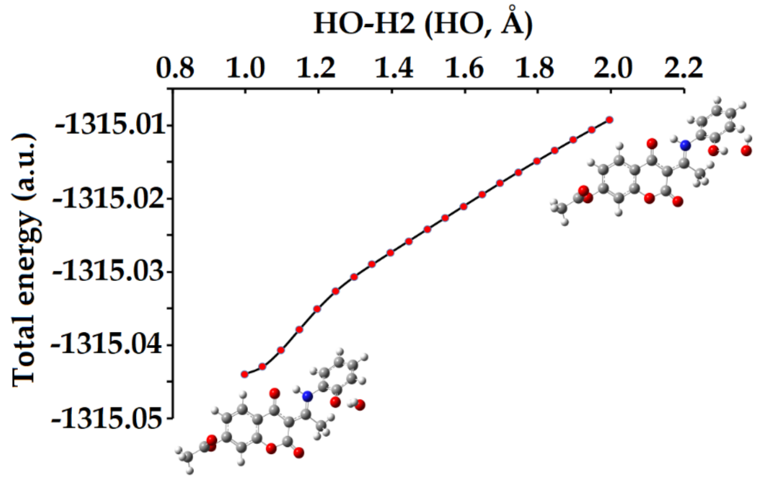

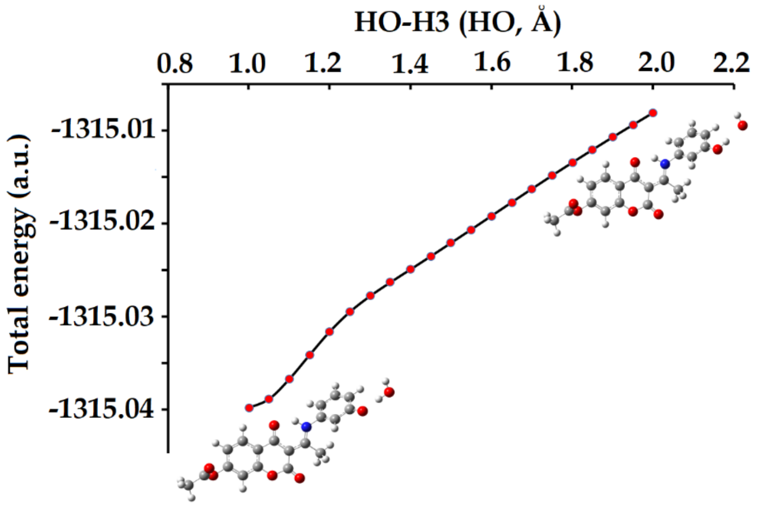

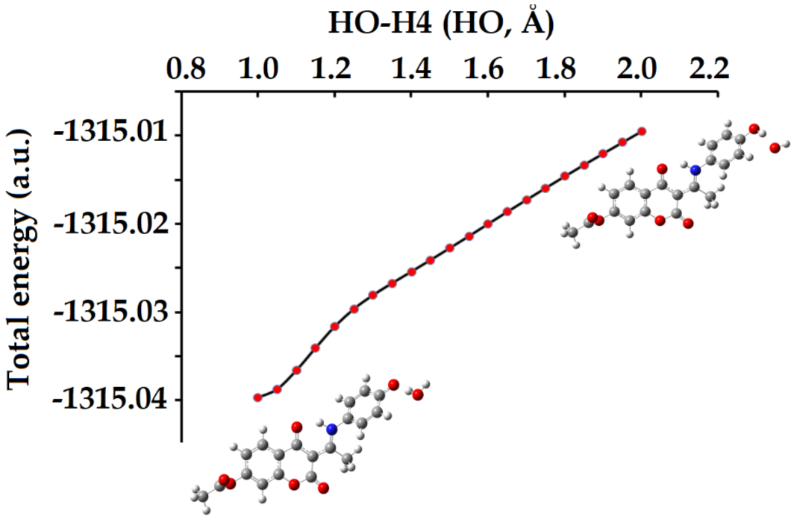

Figure S7. Dependence of total energy (a.u.) on the characteristic HO–H2 (**A_1-RH,** up) HO–H3 (**A_2-RH**, medium) and HO–H4 (**A_3-RH**, down) distance (Å) for the SPL mechanism

Table S2. Estimated overall rate constants ($k_{overall}$) and branching ratios (Γ_i,%) at pH=7.4 for newly synthesized coumarin derivatives **A1-RH** – **A3-RH**

Mechanism	Position	$\Gamma_{A_1\text{-RH}}$ (%)	$\Gamma_{A_2\text{-RH}}$ (%)	$\Gamma_{A_3\text{-RH}}$ (%)
**HAT/PCET**	**–OH**	16.06	15.78	16.20
**RAF**	**C-3**	0.10	0.09	0.01
	**C-5**	0.00	0.00	0.00
	**C-6**	0.00	0.00	0.00
	**C-7**	0.00	0.00	0.00
	**C-8**	0.01	0.02	0.00
	**C-9**	0.00	0.00	0.00
	**C-10**	0.00	0.01	0.00
	**C-1′**	0.00	0.00	0.00
	**C-1″**	0.08	0.00	0.09
	**C-2″**	0.01	0.28	0.00
	**C-3″**	0.07	0.09	0.16
	**C-4″**	0.00	0.87	0.00
	**C-5″**	0.05	0.00	0.16
	**C-6″**	0.02	0.36	0.04
**SPLET**	**-OH**	16.06	15.78	16.20
	**-O⁻**	67.40	66.16	67.00
$k_{overall}^{A1-RH}/k_{overall}^{A2-RH}/k_{overall}^{A3-RH}$		**1.21×10^{10}**	**1.19×10^{10}**	**1.18×10^{10}**

Table S3. Half-life ($\tau_{1/2}$) of investigated compounds (**A1-RH** – **A3-RH**) at physiological pH (7.4) and different concentrations (M) of $HO^{\bullet}$ radical

Compound	10^{-18}	10^{-17}	10^{-6}	10^{-15}	10^{-14}	10^{-10}
	day	*day*	*day*	*h*	*h*	*s*
**A1-RH**	663.0	66.3	6.6	16.8	1.6	0.6
**A2-RH**	674.2	67.4	6.7	16.8	1.6	0.6
**A3-RH**	679.9	68.0	6.8	16.8	1.6	0.6

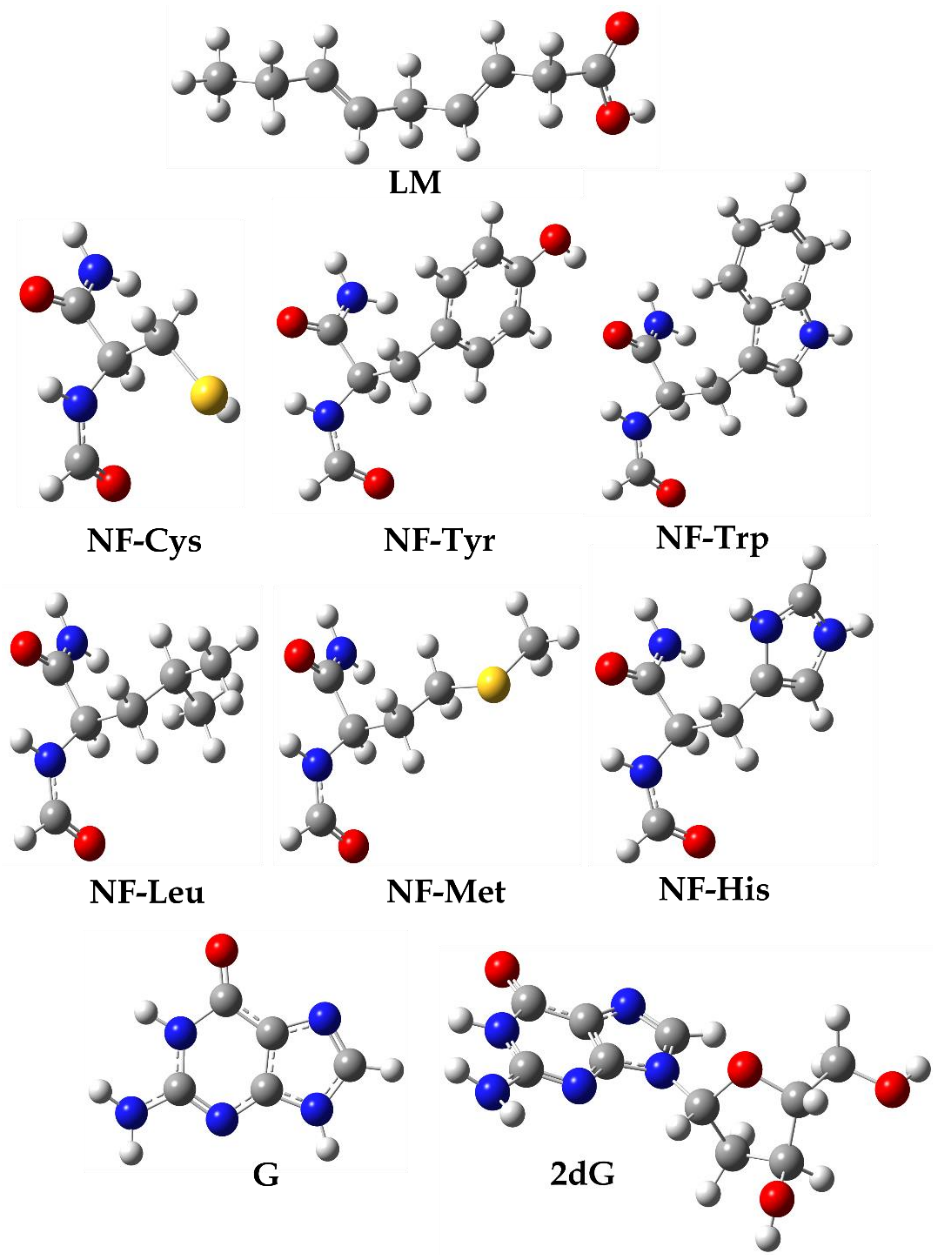

Figure S8. Optimized geometries of selected biomolecular target compounds in water at M06-2X/6-311++G(d,p) level of theory

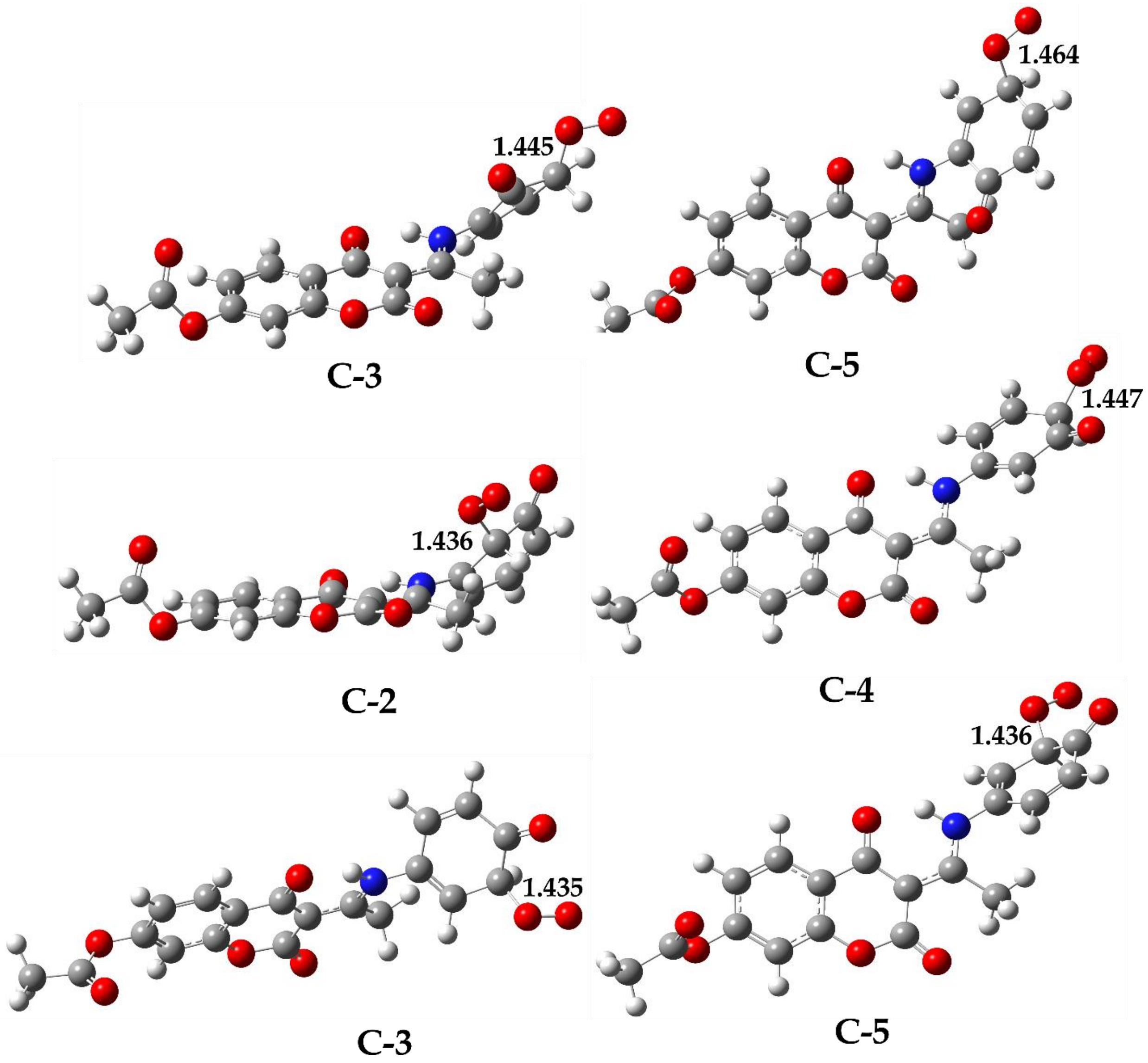

Figure S9. Optimized geometries of intermediate radical adducts, **IN1**, formed in the reaction **A$_1$-RH** (up), **A$_2$-RH** (medium), **A$_3$-RH** (down) and O_2 in water at M06-2X/6-311++G(d,p) level of theory

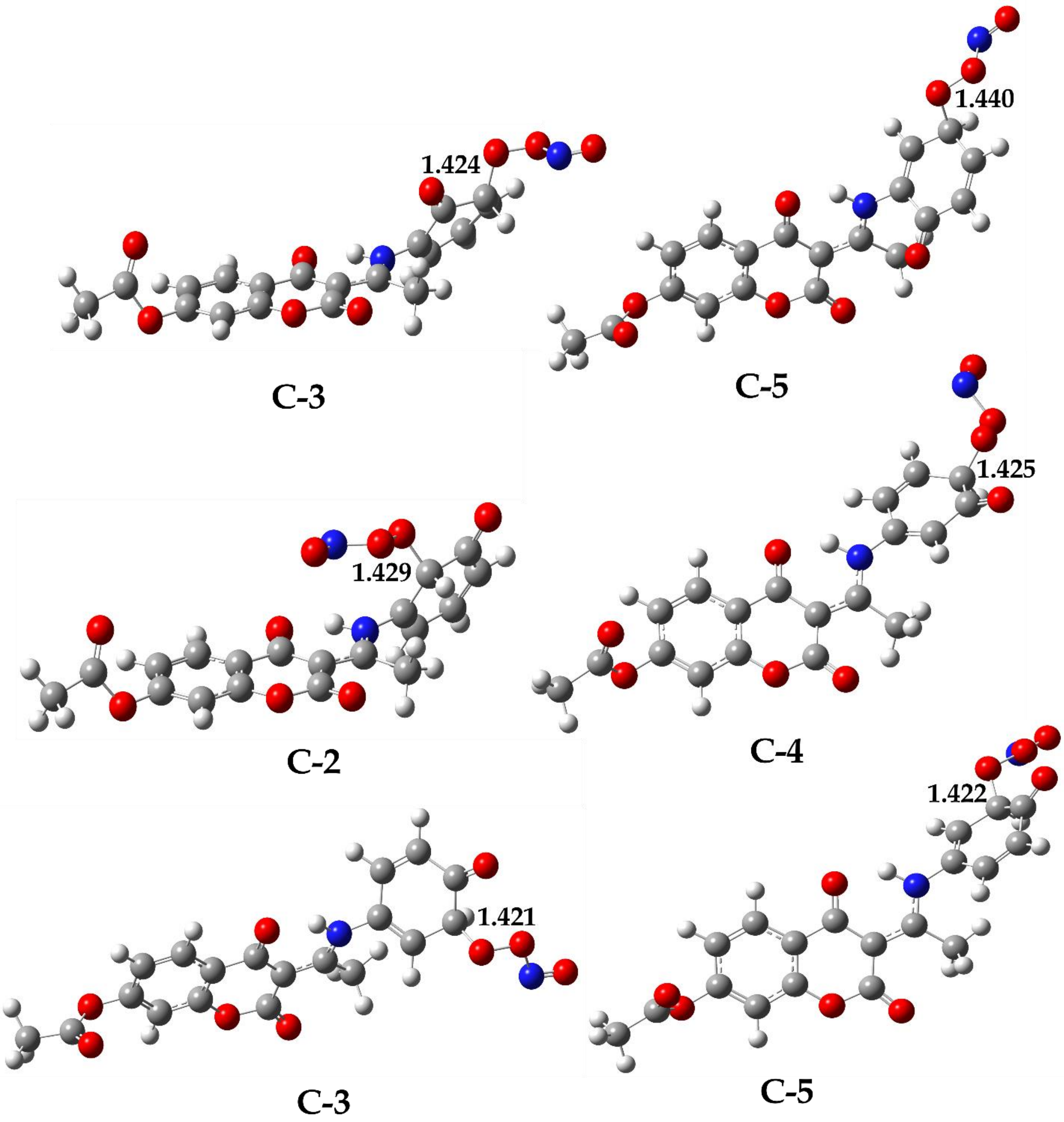

Figure S10. Optimized geometries of intermediate adducts, **P1**, formed in the reaction **A_1-R$^{\bullet}$** (IN1, up), **A_2-R$^{\bullet}$** (IN1, medium), **A_3-R$^{\bullet}$** (IN1, down) and NO in water at M06-2X/6-311++G(d,p) level of theory

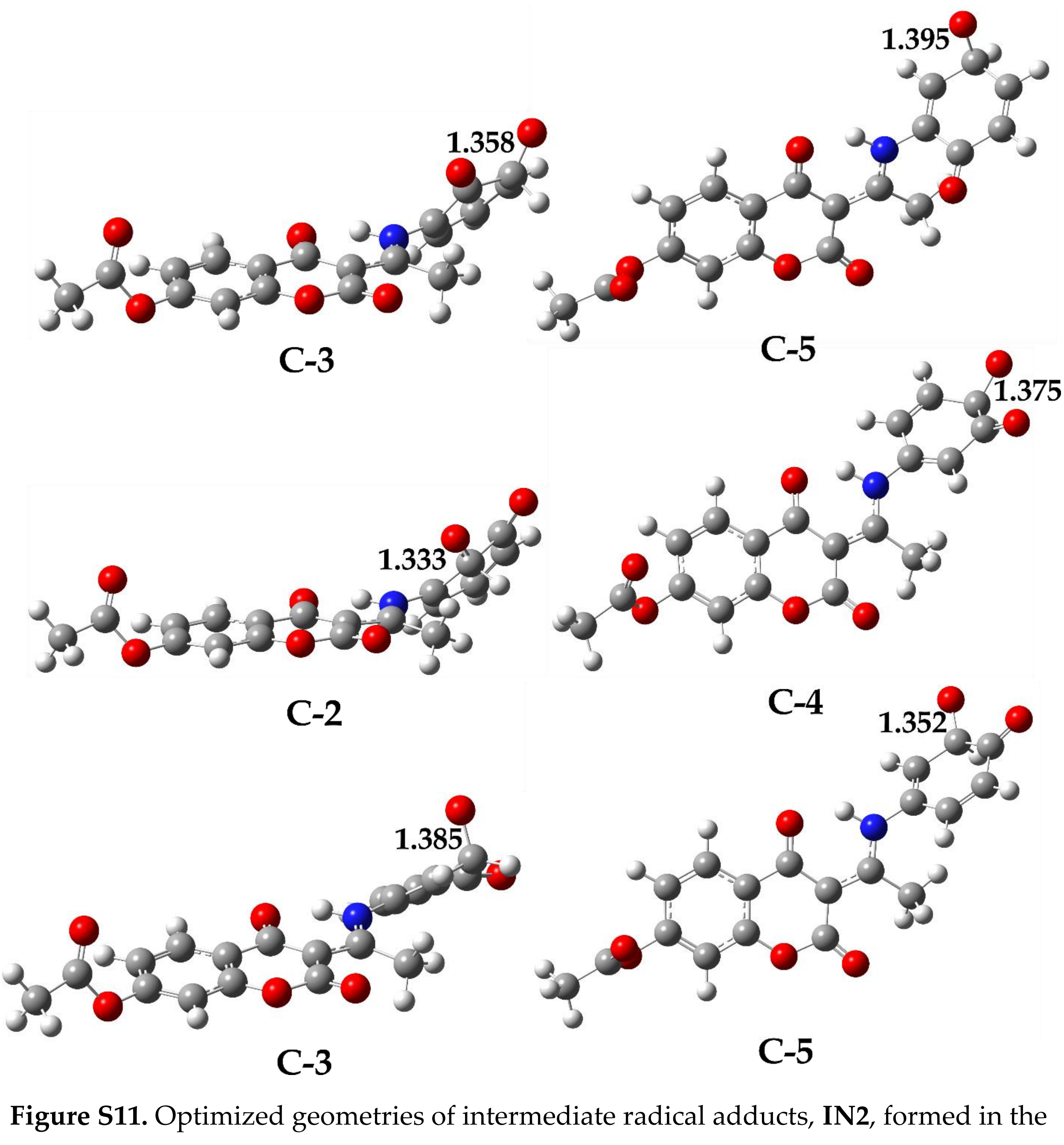

Figure S11. Optimized geometries of intermediate radical adducts, **IN2**, formed in the reaction intramolecular separation NO_2 molecules of **A_1-R˙** (P1, up), **A_2-R˙** (P1, medium), **A_3-R˙** (P1, down) in water at M06-2X/6-311++G(d,p) level of theory

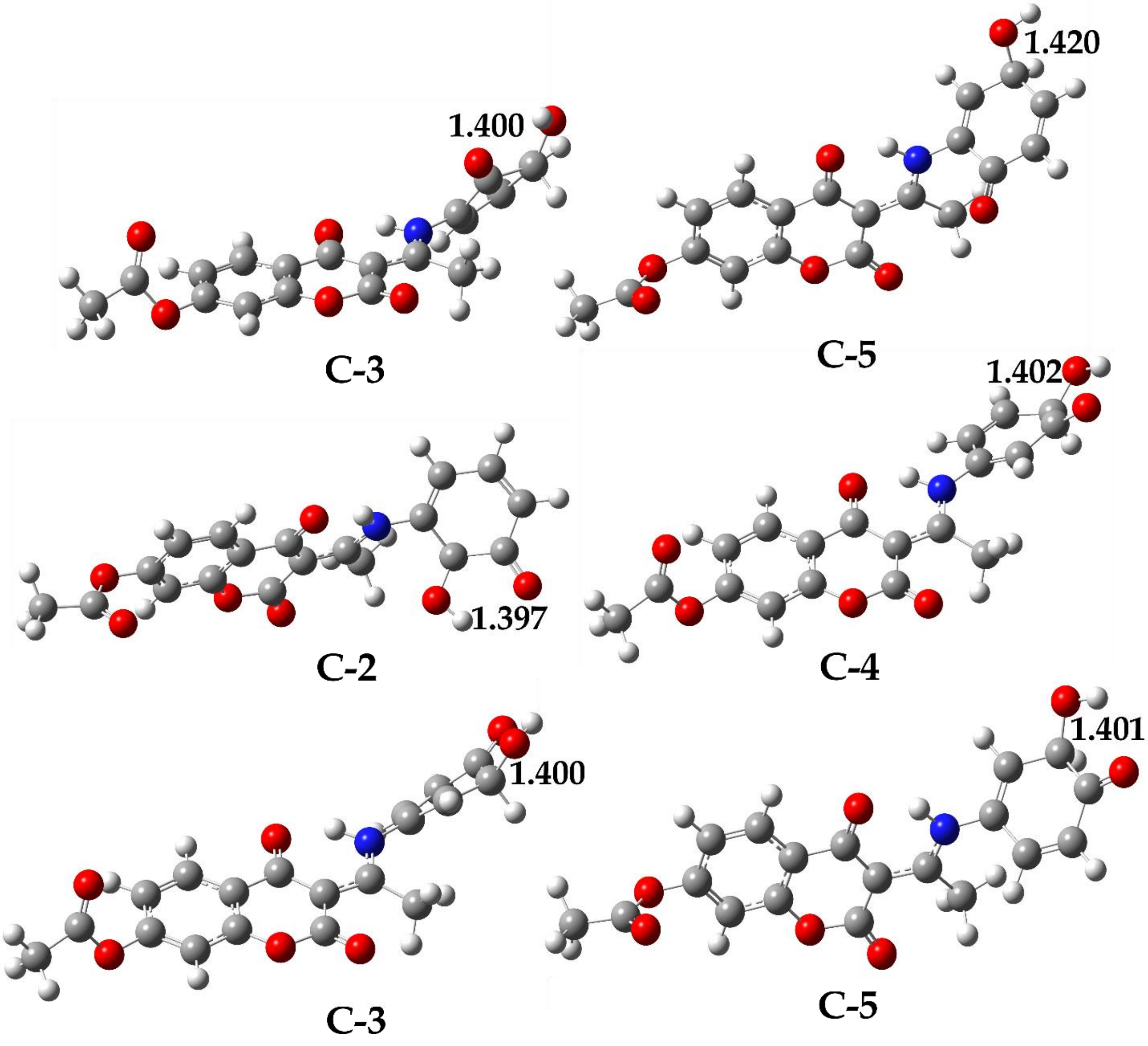

Figure S12. Optimized geometries of intermediate adducts, **P3**, formed in the reaction **A_1-R$^\bullet$** (up), **A_2-R$^\bullet$** (medium), **A_3-R$^\bullet$** (down) and HO$^\bullet$ in water at M06-2X/6-311++G(d,p) level of theory
